# Origin and evolution of *Petrocosmea* (Gesneriaceae) inferred from both DNA sequence and novel findings in morphology with a test of morphology-based hypotheses

**DOI:** 10.1186/s12870-015-0540-3

**Published:** 2015-07-03

**Authors:** Zhi-Jing Qiu, Yuan-Xue Lu, Chao-Qun Li, Yang Dong, James F. Smith, Yin-Zheng Wang

**Affiliations:** State Key Laboratory of Systematic and Evolutionary Botany, Institute of Botany, Chinese Academy of Sciences, 20 nanxincun, Beijing, 100093 China; Key Laboratory of Southern Subtropical Plant Diversity, Fairylake Botanical Garden, Shenzhen & Chinese Academy of Sciences, Shenzhen, 518004 China; Kunming Institute of Botany, Chinese Academy of Sciences, Kunming, 650204 China; Boise State University, Department of Biological Sciences, Boise, ID 83725-1515 USA

**Keywords:** DNA sequence, Evolution, Floral morphology, Gesneriaceae, Himalaya-Tibetan plateau, *Petrocosmea*

## Abstract

**Background:**

*Petrocosmea* Oliver (Gesneriaceae) currently comprises 38 species with four non-nominate varieties, nearly all of which have been described solely from herbarium specimens. However, the dried specimens have obscured the full range of extremely diverse morphological variation that exists in the genus and has resulted in a poor subgeneric classification system that does not reflect the evolutionary history of this group. It is important to develop innovative methods to find new morphological traits and reexamine and reevaluate the traditionally used morphological data based on new hypothesis. In addition, *Petrocosmea* is a mid-sized genus but exhibits extreme diverse floral variants. This makes the genus of particular interest in addressing the question whether there are any key factors that is specifically associated with their evolution and diversification.

**Results:**

Here we present the first phylogenetic analyses of the genus based on dense taxonomic sampling and multiple genes combined with a comprehensive morphological investigation. Maximum-parsimony, maximum likelihood and Bayesian analyses of molecular data from two nuclear DNA and six cpDNA regions support the monophyly of *Petrocosmea* and recover five major clades within the genus, which is strongly corroborated by the reconstruction of ancestral states for twelve new morphological characters directly observed from living material. Ancestral area reconstruction shows that its most common ancestor was likely located east and southeast of the Himalaya-Tibetan plateau. The origin of *Petrocosmea* from a potentially *Raphiocarpus*-like ancestor might have involved a series of morphological modifications from caulescent to acaulescent habit as well as from a tetrandrous flower with a long corolla-tube to a diandrous flower with a short corolla-tube, also evident in the vestigial caulescent habit and transitional floral form in clade A that is sister to the remainder of the genus. Among the five clades in *Petrocosmea*, the patterns of floral morphological differentiation are consistent with discontinuous lineage-associated morphotypes as a repeated adaptive response to alternative environments.

**Conclusion:**

Our results suggest that the lineage-specific morphological differentiations reflected in the upper lip, a functional organ for insect pollination, are likely adaptive responses to pollinator shifts. We further recognize that the floral morphological diversification in *Petrocosmea* involves several evolutionary phenomena, i.e. evolutionary successive specialization, reversals, parallel evolution, and convergent evolution, which are probably associated with adaptation to pollination against the background of heterogeneous abiotic and biotic environments in the eastern wing regions of Himalaya-Tibetan plateau.

**Electronic supplementary material:**

The online version of this article (doi:10.1186/s12870-015-0540-3) contains supplementary material, which is available to authorized users.

## Background

In current plant systematics, research activity tends to begin with phylogenetic reconstruction based on DNA sequence data. Molecular systematics has revolutionized traditional plant systematics and classification. However, the morphological support for such changes has often been absent, or consisted of ad hoc explanations. In many cases, the few morphological characters used to support molecular phylogenies are selected from the characters that were used to initially describe the taxa, rather than novel characters from active morphological and anatomical research. This situation is mainly due to the misunderstanding that everything in morphology has been completed [1]. On the contrary, numerous morphological characters are yet unexplored, especially in tropical groups. Many of these characters may reflect the evolutionary histories of these taxa and serve as a complement to molecular phylogenies.

*Petrocosmea* Oliv. (Gesneriaceae, Didymocarpoideae sensu Weber *et al.* 2013) [2] contains 38 species with four non-nominate varieties, all mostly distributed in southwestern China with several species in Northern Myanmar and Thailand, and Northeastern India [3–6]. The genus has been divided into three subgeneric sections. Hemsley (1899) [7] erected section *Anisochilus* Hemsl. because two species, *P. iodioides* Hemsl. and *P. minor* Hemsl., have an upper lip that is much shorter than the lower lip making them distinctive from *P. sinensis Oliv..* Craib (1919) [8] made the first revision of the genus with 15 species placing them in sections *Petrocosmea* Craib and *Anisochilus*. In the second revision that included 27 species and four varieties, Wang (1985) [9] principally followed Craib (1919) [8] but established sect. *Deinanthera* W. T. Wang. Members of this latter section have anthers constricted near the apex that create a short thick beak. Wang’s classification system has been followed by later authors [3–5].

Few morphological characters were utilized in the sectional divisions and species descriptions, probably because most information was lost on dried specimens. For example, the subgeneric rankings were roughly based on the length ratios of the upper lip (two upper corolla lobes) to the lower lip (two lateral and one lower corolla lobes), and the degree of fusion of the two upper corolla lobes [3–5, 8, 9]. From the description of different sections and species, it would appear that the flowers are morphologically simple in *Petrocosmea*.

**Fig. 1:**
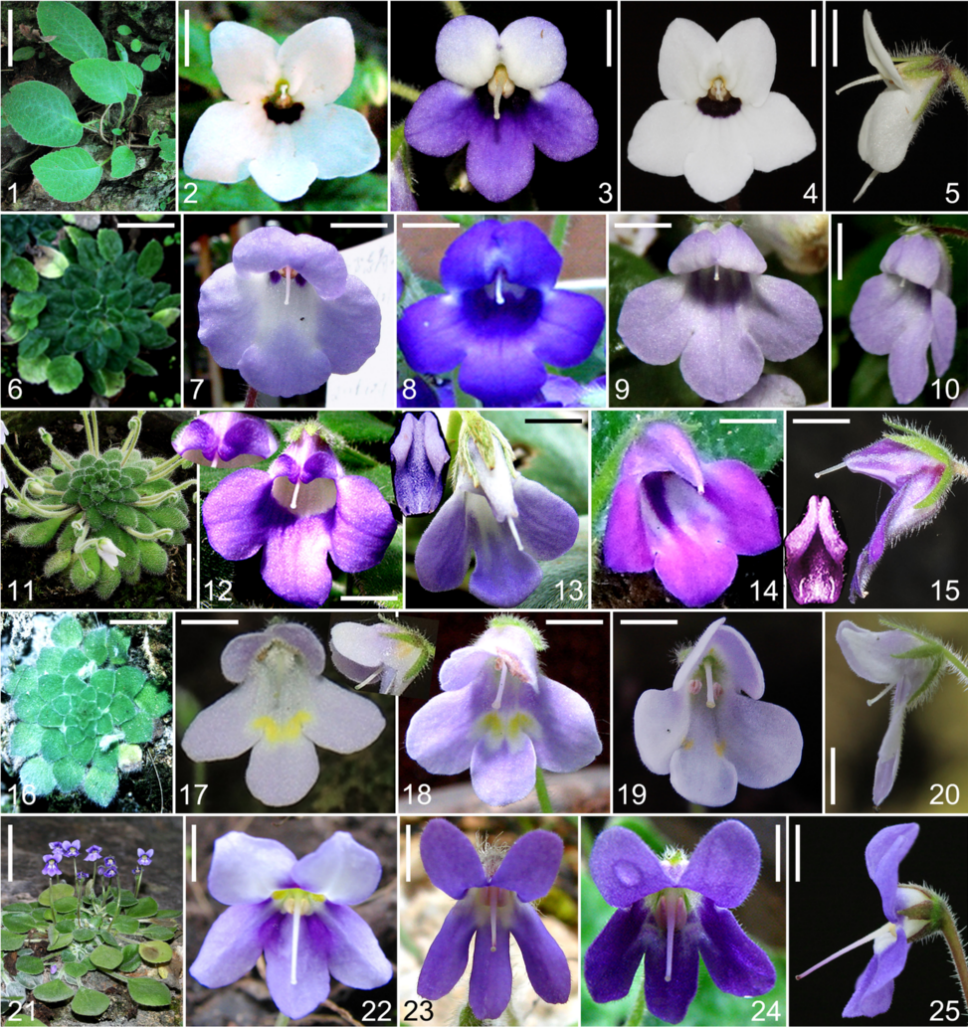
Photos of representative habits and flowers of different clades. 1-5 (clade A): 1. habit of *P. menglianensis*, showing leaves erect; 2-5. flowers of *P. menglianensis* (2), *P. kerrii* (3) and *P. grandifolia* (4-5). Scale bars = 6 cm (1), 6 mm (2), 2.8 mm (3), 5.6 mm (4) and 4.2 mm (5). 6-10 (clade B): 6. habit of *P. mairei var. intraglabra*, showing leaves arranged in basal rosettes spreading on the ground; 7-10. flowers of *P. duclouxii* (7), *P. coerulea* (8) and *P. mairei var. intraglabra* (4-5), note upper lips reflexed backward. Scale bars = 2.5 cm (6), 4.5 mm (7) and 5 mm (8-10). 11-15 (clade C): 11. habit of *P. minor*, showing leaves arranged in basal rosettes spreading on the ground; 12-15. flowers of *P. iodioides* (12), *P. sericea* (13) and *P. minor* (14-15), showing the carinate-plicate (galeate) structure of the upper lip. Scale bars = 2.1 cm (11), 3.6 mm (12) (upper lip closed up to 1.45 times), 4.2 mm (13) (upper lip closed up to one time), 4.1 mm (14) and 3 mm (15) (upper lip closed up to one time). 16-20 (clade D): 16. habit of *P. forrestii*, showing leaves arranged in basal rosettes spreading on the ground, 17-20. flowers of *P. barbata* (17), showing cilia (hairs) on inner side of corolla tube and the upper lip extended forward, *P. mairei* (18) and *P. forrestii* (19-20), showing upper lips extended forward. Scale bars = 1.5 cm (16), 4.5 mm (17), 4.4 mm (18), 3.2 mm (19) and 2.9 mm (20). 21-25 (clade E): 21. habit of *P. sinensis*, showing leaves arranged in basal rosettes spreading on the ground; 22-25. flowers of *P. oblata* (22), *P. nervosa* (23) and *P. sinensis* (24-25). Scale bars = 3 cm (21), 4.7 mm (22), 5.6 mm (23), 5.3 mm (24) and 4.1 mm (25). Note: The upper lips (two upper corolla lobes) are arranged above and the lower lips (three lower corolla lobes) below in all flowers

In reality, the flowers of *Petrocosmea* are morphologically extremely varied, but much of this variation is not reflected in the present classification. For example, section *Anisochilus* Hemsl. is traditionally defined by a length ratio of 1:2 between the upper and lower lips. Three groups of species within this section are distinctively different in the morphology of the upper lip even though they have the similar upper lip lengths. The first group is characterized by the upper lip reflexed backward while the second group has the upper lip extended forward with a flat surface (Fig. 1 clades B and D). Meanwhile, the upper lip of the third group has a specialized morphological structure that has not been observed in other species of *Petrocosmea*; the two upper corolla lobes extend forward and are fused nearly their full length with each lobe folded and rolled laterally to form a carinate-plicate structure (Fig. 1 clade C). This carinate-plicate structure of the upper lip encloses the style which is pressed against the inner surface to establish a complex structure with unknown biological function. These specific morphological structures of the three groups in section *Anisochilus* are correlated with other morphological variations (for details see Results). This morphological variation is lacking in the traditional descriptions of *Petrocosmea* and cannot easily be observed in dried specimens. Therefore, it is doubtful that the similarity in length ratios of the upper to lower lips is homologous among species in section *Anisochilus*. Likewise other morphological characters traditionally utilized in the classification of *Petrocosmea* are unlikely to be homologous. As Darwin pointed out “No group of organic beings can be well understood until their homologies are made out” [10]. The recognition of homology is the first step to reconstruct the morphological relationships and evolutionary trends in any plant group.

Since *Petrocosmea* was describecd [11], no molecular systematic study has focused on the phylogeny of *Petrocosmea* except for a few species that have been sampled in molecular phylogenetics at higher ranks in Gesneriaceae [12–15]. A phylogenetic reconstruction based on DNA sequence data from multiple loci would enhance our understanding of morphological diversity in relation to evolutionary history and test the interpretation of morphological evolution and homology in this genus. In addition, the presently distributed area of *Petrocosmea* in the northern Myanmar and Thailand, northeastern India and southwestern China is just located in the eastern wing region of the Himalaya-Tibetan plateau. This is where the Hengduan Mountains, that consist of rugged terrain with high mountains alternating with several deep gorges, runs parallel north to south. The Hengduan Mountains have not only been widely considered an important center of survival, but also a well-known region of speciation and evolution in the world [16, 17]. It would be interesting to know whether the origin and diversification of *Petrocosmea* are related to this heterogeneous ecogeographical environment.

In the present study, we analyzed a multi-gene dataset including two nuclear (*ITS*, *Petrocosmea CYCLOIDEA1D* (*PeCYC1D*)) and six plastid regions (*atpI-H, matK, trnH-psbA, rps16, trnL-trnF, trnT-trnL*) from 35 species and three non-nominate varieties of *Petrocosmea* with two species of *Raphiocarpus* plus an additional species of *Boea* and two species of *Streptocarpus* as outgroups. Since we had continuously carried out field observation and had collected living plants of most *Petrocosmea* species in the greenhouse, we also conducted a comprehensive investigation on the flower morphology of *Petrocosmea* by dissecting the flower into a series of units for detailed comparison and analyses. Forty-one morphological characters from both vegetative and floral organs were analyzed to reconstruct the relationship within *Petrocosmea* alone and combined with molecular data. Our objectives in this study are (1) to test the monophyly of the genus; (2) to explore the morphological origin and differentiation pattern of *Petrocosmea*; (3) to interpret the evolutionary significance of the morphological differentiation within a robust phylogenetic context linked to both biotic and abiotic environment; and finally (4) to evaluate the newly observed vs. traditionally utilized morphological characters in relation to the role of morphological data in phylogenetic reconstruction.

## Results

### Analyses of DNA sequence and morphological data separately

The combined cpDNA matrix, which comprises six chloroplast regions of *trnL-F*, *matK*, *rps16*, *atpI-atpH*, *trnH-psbA*, and *trnT-L*, had aligned sequences of 5662 bp, of which 4719 (83.35 %) were constant, 560 (9.89 %) were variable but uninformative, and 383 (6.76 %) were parsimony informative. We were unable to amplify cpDNA regions from *P. confluens*. Modeltest indicated GTR + G as the best-fit model for the cpDNA sequence data. The strict consensus of 6 trees yielded by MP (Maximum Parsimony) analysis (L = 1182, CI = 0.884, RI = 0.873) was generally congruent with the ML (Maximum Likelihood) tree and the majority rule BI (Bayesian Inference) tree in the topology (Additional file 1: Figure S2). Support values less than 50 % are marked with asterisk.

In the nuclear DNA analysis with *P. confluens* added to the matrix, the ILD (incongruence length different) test gave a p value of 0.42, indicating that the sequence data from ITS and *PeCYC1D* were congruent. The combined nuclear DNA matrix of ITS and *PeCYC1D* consisted of 1662 bp, of which 1213 (72.98 %) were constant, 228 (13.72 %) were variable but uninformative, and 221 (13.3 %) were parsimony informative. Modeltest indicated GTR + G as the best-fit model for the combined nuclear DNA data. The strict consensus of eight trees from MP analysis (L = 642, CI = 0.872, RI = 0.849) was congruent with the ML tree and the majority rule consensus BI tree (Additional file 1: Figure S3).

**Fig. 2:**
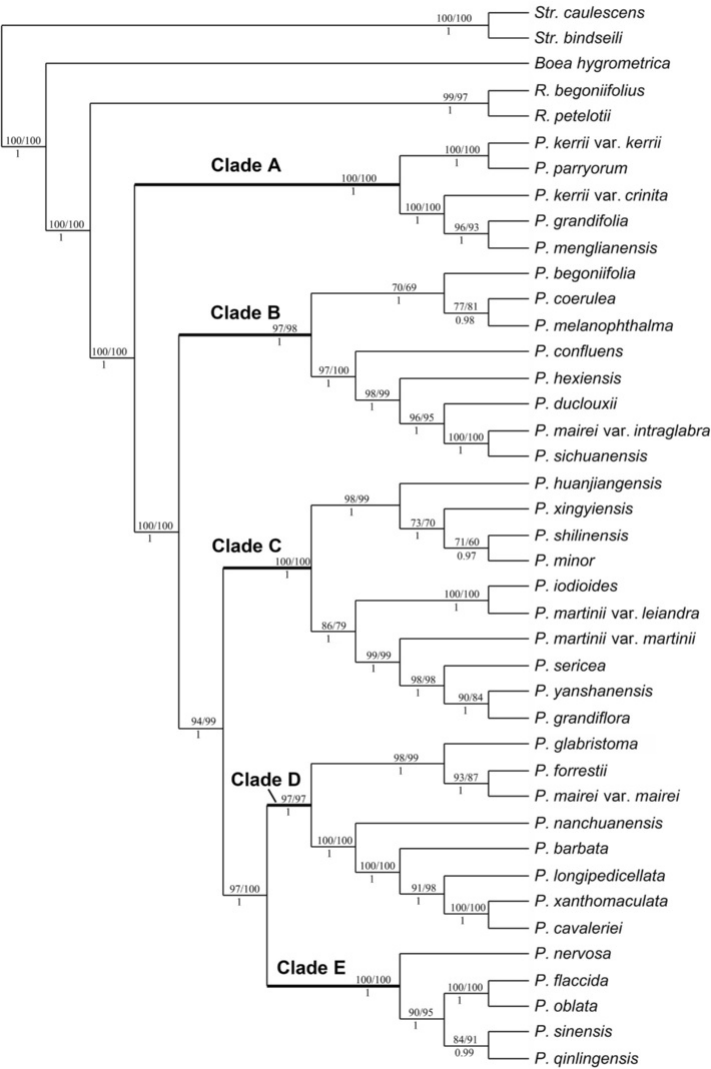
The majority rule consensus Bayesian tree generated from analysis of combined cpDNA and nDNA data. Bootstrap values from MP/ML are shown above branches and posterior probabilities from BI are shown below branches. P. *Petrocosmea*, R. *Raphiocarpus*, Str. *Streptocarpus*

In the combined cpDNA and nuclear DNA analysis, *P. rosettifolia* and *P. longianthera* were removed because of their obvious topological differences between cpDNA and nuclear DNA data, but *P. confluens* was included despite lacking cpDNA data. The ILD test gave a value of p = 0.25, indicating that the data from the two distinct genome regions excluding these two species did not contain significant incongruence. Modeltest suggested that the GTR + G model best fit the combined data. The combined datasets consisted of 7320 bp, 774 (10.57 %) of which were variable and 587 (8.02 %) parsimony informative sites. Parsimony analyses resulted in a single tree (L = 1767, CI = 0.886, RI = 0.872) which was congruent with the ML tree and the majority rule consensus BI tree (Fig. 2).

The MP-ML-BI tree of the combined cpDNA and nuclear DNA datasets was similar to the cpDNA and nuclear DNA trees but with stronger support (Figs. 2, Additional file 1: Figure S2-S3). The combined cpDNA and nuclear DNA tree comprises five main clades labeled A–E (Fig. 2). Each clade receives strong or maximum support, and they are grouped together successively by strong to maximum support (Fig. 2).

For the analysis of the morphological data, Forty-one morphological characters were coded. The strict consensus of 125 trees yielded from the MP analysis (L = 82, CI = 0.842, RI = 0.972) was congruent with the majority rule consensus BI tree (Additional file 1: Figure S4). Similar to the DNA trees, the morphological tree comprises five major clades including the same species as the molecular based trees. However, most nodes within the major five clades have weak to moderate support with frequent polytomies.

### Analysis of combined DNA sequence and morphological data

**Fig. 3:**
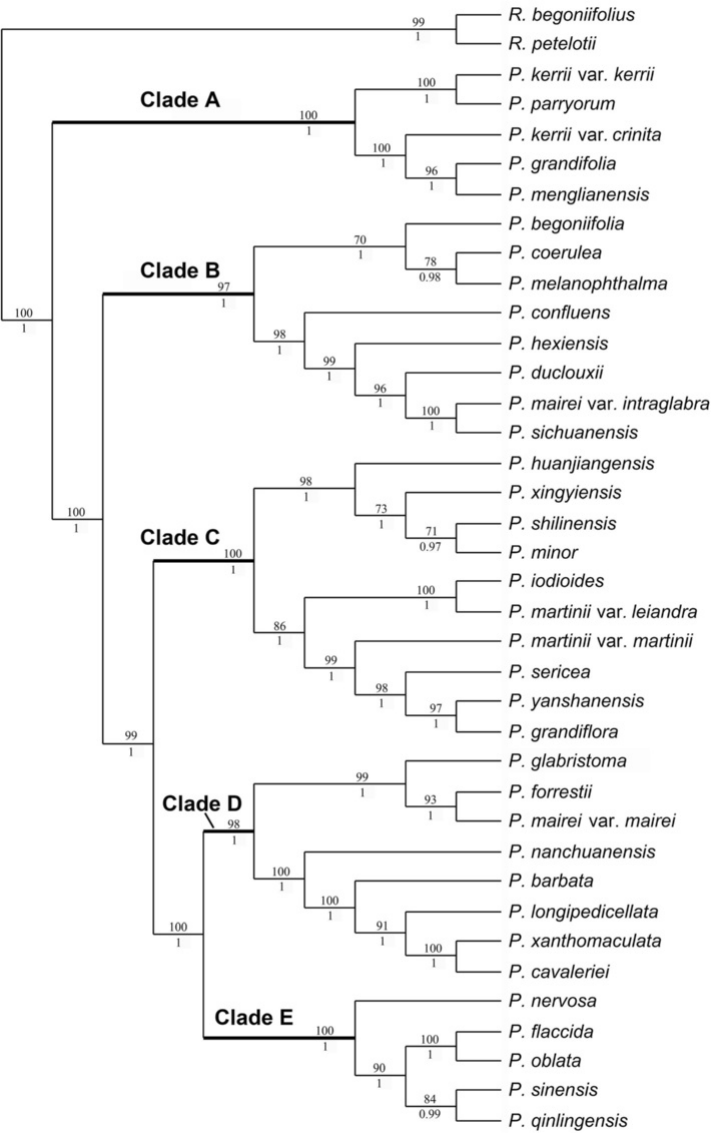
Single most parsimonious trees generated from analysis of combined DNA and morphological data. Note. Bootstrap values from MP are shown above branches and posterior probabilities from BI are shown below branches. P. *Petrocosmea*, R. *Raphiocarpus*

In the analysis of the combined data of DNA and morphology with *P. rosettifolia* and *P. longianthera* removed, the ILD test gave a value of p = 0.082, indicating that the data from the DNA and morphological data did not contain significant incongruence. Both *P. rosettifolia* and *P. longianthera* were removed from the combined molecular and morphological analyses due to the discrepancies in the placement of these two species with ITS and cpDNA. The combined data sets consisted of 7361 bp, 774 (10.51 %) of which were variable and 628 (8.53 %) parsimony informative sites. Parsimony analyses resulted in a single tree (L = 1853, CI = 0.882, RI = 0.888) which was congruent with the majority rule consensus BI tree (Fig. 3).

**Fig. 4:**
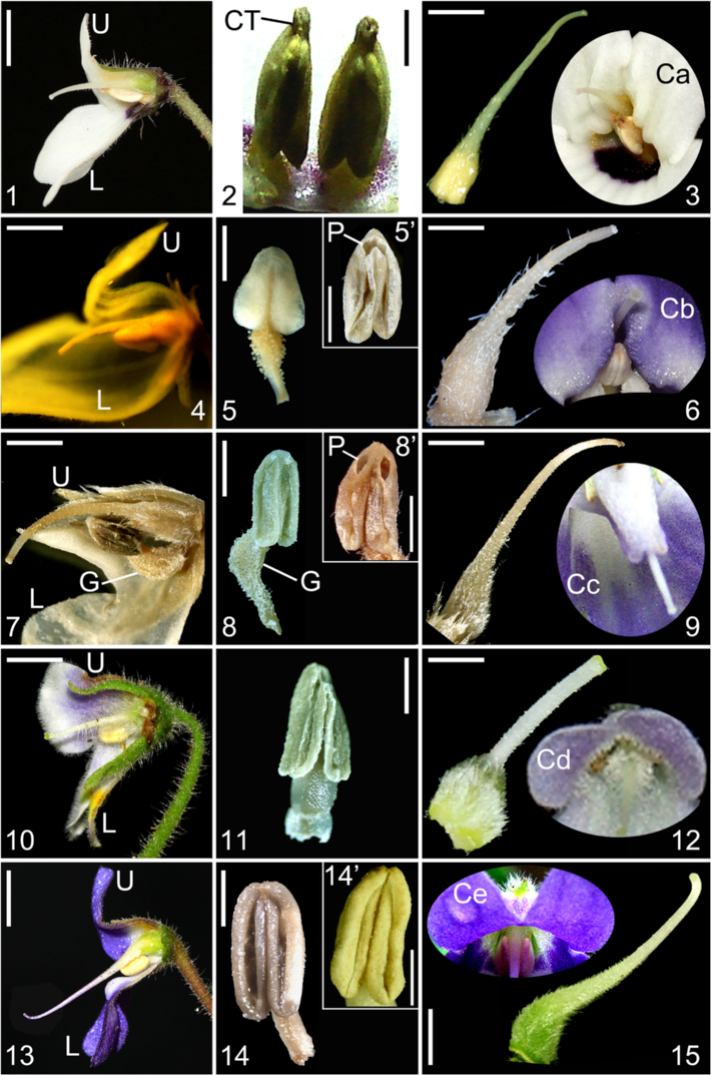
Photos of dissected flowers of representative species of different clades. 1-3 (clade A, *P. grandifolia*): 1.longitudinal section, showing relative position of stamen and pistil inside corolla tube, 2. anthers, showing anthers constricted at top, poricidal with short filament, 3. pistil, showing style’s tip curving downward, Ca showing corolla throat ribbed at both upper and lower sides and relative position of style at throat magnified 1.7 times in size relative to Fig. 1-2. Scale bars = 2.7 mm (1), 1.4 mm (2) and 2.2 mm (3) (3.5 mm in Ca). 4-6 (clade B, *P. mairei var. intraglabra*): 4. longitudinal section, 5. Stamen, showing poricidal anther basifixed with straight filament (5’. anther of *P. coerulea* showing dehiscent pore), 6. Pistil, Cb showing corolla throat of *P. coerulea* ribbed at upper side and relative position of style at throat magnified 2.5 times in size relative to Fig. 1-8. Scale bars = 3.4 mm (4), 1.1 mm (5) (0.86 mm in 5’) and 1.3 mm (6) (2 mm in Cb). 7-9 (clade C, *P. sericea*): 7. longitudinal section, 8. Stamen, showing poricidal anther basifixed with long geniculate filament (8’. anther of *P. minor* showing dehiscent pore), 9. pistil, showing style curving downward at top, Cc showing corolla throat unribbed and relative position of style at throat magnified 2 times in size relative to Fig. 1-13. Scale bars = 2.9 mm (7), 1.8 mm (8) (1.6 mm in 8’) and 2.2 mm (9) (2.1 mm in Cc). 10-12 (clade D, *P. forrestii* and *P. barbata*): 10. longitudinal section of *P. barbata*, showing style extending from centre of the throat, 11. Stamen of *P. forrestii*, showing anther longitudinal dehiscent with short filament, 12. Pistil of *P. forrestii*, erect, Cd showing corolla throat of *P. barbata* unribbed and relative position of style at throat magnified 1.75 times in size relative to Fig. 1-17. Scale bars = 3.6 mm (10), 1.3 mm (11) and 1.9 mm (12) (2.6 mm in Cd). 13-15 (clade E, *P. sinensis*): 13. longitudinal section, 14. Stamen, showing longitudinal anther with short filament (14’. showing anther with longitudinal dehiscence becoming visible), 15. pistil, showing style curving downward at the base and curving upward at the top, Ce showing corolla throat unribbed and relative position of style at throat magnified 1.26 times in size relative to Fig. 1-24. Scale bars = 3.7 mm (13), 1.4 mm (14 and 14’) and 2.9 mm (15) (4.2 mm in Ce). CT, constriction; G, geniculate; L, lower lip; P. dehiscent pore; U, upper lip

The trees of the combined data set of DNA and morphology and the combined DNA data are identical in topology with only a few fluctuations in support values of some branches (Figs. 2-3). The tree of combined DNA and morphological data consists of five major clades labeled A-E with strong to maximum support, which are clustered together with maximum support (Fig. 3). Clade A, which consists of four taxa (*P. kerrii* var. *kerrii*, *P. kerrii* var. *crinita*, *P. menglianensis*, and *P. grandifolia*) of sect. *Deinanthera* sensu Wang (1985) [9] and one species (*P. parryorum*) of sect. *Anisochilus* sensu Wang (1985) [9], is sister to the remaining species with maximum support. The five species bear a series of synapomorphies exclusive to clade A, i.e., vestigial caulescent habit with ascendant leaves, an upper lip slightly shorter than the lower lip in length, anthers that are constricted at the tip and two dark red-brown spots on the lower side of the corolla-tube below the filaments (Figs. 1, 4). In addition, *P. kerrii* var. *kerrii* is sister to *P. parryorum* with maximum support*,* a relationship that is morphologically reflected in the shared feature of blue-violet flowers with geniculate filaments. In contrast, *P. kerrii* var. *crinita* is sister to *P. grandifolia*/*P. menglianensis* with maximum support rather than sister to the type variety of *P. kerrii*, consistent with their shared traits of white flowers with straight filaments. *Petrocosmea kerrii* var. *kerrii* and *P. kerrii* var. *crinita* are apparently two independent species because they are not recovered as an exclusive monophyletic group.

Clade B contains eight taxa (*P. coerulea*, *P. begoniifolia*, *P. melanophthalma*, *P. confluens*, *P. hexiensis*, *P. duclouxii*, *P. sichuanensis*, and *P. mairei* var. *intraglabra*) of sect. *Anisochilus* sensu Wang (1985) [9], and is a well-supported clade sister to clades C-D with maximum support. *Petrocosmea mairei* var. *intraglabra* and *P. sichuanensis* as a pair of sister species with maximum support are strongly supported to come together successively with *P. duclouxii* (MP-BS (bootstrap) =96 %; PP (posterior probabilities) =100 %), *P. hexiensis* (MP-BS = 99 %; PP =100 %), and *P. confluens* (MP-BS = 98 %; PP = 100 %). *Petrocosmea coerulea* and *P. melanophthalma* as sister species with moderate support (MP-BS = 78 %; PP = 98 %) are further clustered together with *P. begoniifolia* with MP-BS = 70 % and PP = 100 %). The two branches in clade B are further joined together with strong support (MP-BS = 97 %; PP = 100 %). The species of clade B are defined by their short upper lips with semiorbicular corolla lobes. The morphological synapomorphies of clade B also include two upper corolla lobes highly reflexed backward with two purple spots on the lower side of the corolla-tube below the filaments (Fig. 1). Apparently, *P. mairei* var. *intraglabra* is a species apart from *P. mairei* var. *mairei* which is nested in clade D (Figs. 2-3).

Clade C includes eight taxa (*P. iodioides*, *P. martinii* var. *leiandra*, *P. martinii* var. *martinii*, *P. minor*, *P. sericea*, *P. shilinensis*, *P. xingyiensis* and *P. huanjiangensis*) of sect. *Anisochilus* and two species (*P. grandiflora* and *P. yanshanensis*) of sect. *Petrocosmea*. There are two lineages in Clade C with maximum support. In one lineage, *P. grandiflora* and *P. yanshanensis* as strongly supported sister species (MP-BS = 97 %; PP = 100 %) are grouped in sequence with *P. sericea* (MP-BS = 98 %; PP = 100 %), *P. martinii* var. *martini* (MP-BS = 99 %; PP = 100 %), and maximally supported sister species of *P. iodioides* and *P. martinii* var. *leiandra*. In another lineage, *P. minor* and *P. shilinensis* are sister to each other (MP-BS = 71 %; PP = 97 %), and further grouped with *P. xingyiensis* by moderate support (MP-BS = 73 %; PP = 100 %), and together they are sister to *P. huanjiangensis* with strong support (MP-BS = 98 %; PP = 100 %).

**Fig. 5:**
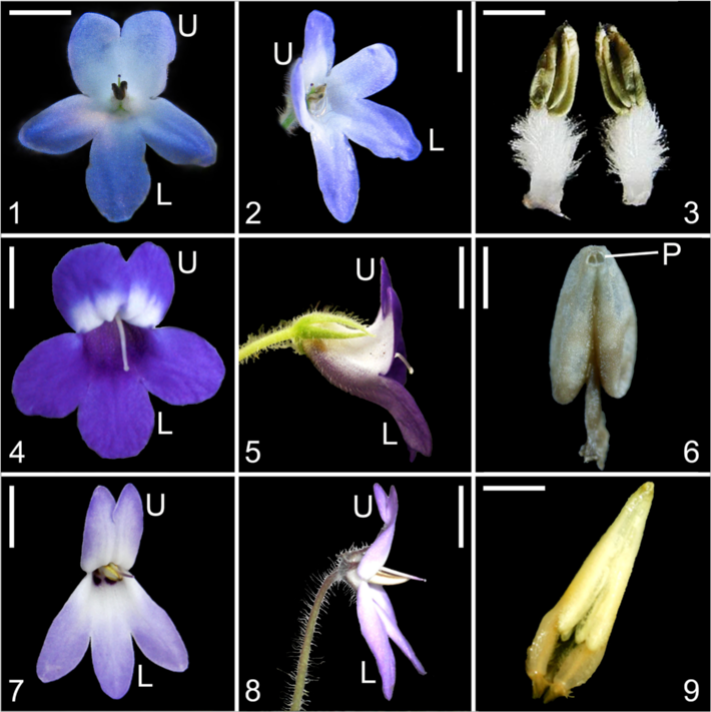
Photos of flowers of *P. yanshanensis*, *P. rosettifolia* and *P. longianthera*. 1-3. *P. yanshanensis*: face view (1), lateral view (2) and stamens (3); 4-6. *P. rosettifolia*: face view (4), lateral view (5) and stamen indicating poricidal anther (6); 7-9. *P. longianthera*: face view (7), lateral view (8) and stamens indicating long anthers with short filaments (9). Scale bars = 5.7 mm (1-2), 1.4 mm (3), 6 mm (4), 5.6 mm (5), 1.6 mm (6), 5.4 mm (7-8) and 1.8 mm (9). L, lower lip; P, dehiscent pore; U, upper lip

The eight species traditionally placed in sect. *Anisochilus* all share a specific floral character; the two upper corolla lobes are fused nearly their entire length and each lobe is folded and rolled laterally to form a carinate-plicate shape of the upper lip that encloses the style. In the traditional classification, the upper lip of these species is only described by the phrase “indistinctly 2-lobed, emarginate, or undivided”. This specific structure of the upper lip is first recognized herein in *Petrocosmea* (Fig. 1). *Petrocosmea grandiflora* and *P. yanshanensis* as a pair of sister species exhibit a series of floral characters distinctively different from other species of clade C (Fig. 5). These two species have striking similarities to species of clade E in the external appearance of the corolla (Fig. 5), the reason that they all had been formerly placed in sect. *Petrocosmea*. Nevertheless, the highly fused upper lips in the flowers of *P. grandiflora* and *P. yanshanensis* as the synapomorphy shared with other species of clade C hint at membership in clade C. The similarity between these two species and members of clade E is likely the result of floral convergent evolution. Clade C is sister to clades D and E with maximum support.

Clade D comprises six taxa (*P. forrestii*, *P. mairei* var. *mairei*, *P. barbata*, *P. cavaleriei*, *P. xanthomaculata*, and *P. longipedicellata*) of sect. *Anisochilus* and two newly described species *P. nanchuanensis* and *P. glabristoma* with strong support (MP-BS = 98 %; PP = 100 %). *Petrocosmea nanchuanensis* is sister to a maximally supported branch containing *P. barbata*, and *P. longipedicellata* gathered together by strong support (MP-BS = 91 %; PP = 100 %) with two maximally supported sister species, *P. cavaleriei* and *P. xanthomaculata*. These five species as a maximum supported branch are further united with three well resolved sister species *P. glabristoma*, *P. forrestii* and *P. mairei* var. *mairei*. The species in clade D have a generally similar bilateral corolla to the species in clade B. However, the two lobes in the upper lip are extended forward rather than reflexed backward. In addition, they can also be easily recognized by two bright yellow spots or cicatrices on the lower lip and hairs on the upper lip in the corolla throat (Fig. 1).

Five species (*P. nervosa*, *P. oblata*, *P. flaccida*, *P. sinensis*, and *P. qinlingensis*) of sect. *Petrocosmea* form clade E with maximum support. In clade E, *P. oblata* and *P. flaccida* are sister with maximum support and these two are grouped with another set of sister species, *P. sinensis* and *P. qinlingensis,* with strong support (MP-BS = 90 %; PP = 100 %). *Petrocosmea nervosa* is sister to the remaining species in Clade E with maximum support. The species of clade E all share a large bilobed upper lip that is equal or almost equal to the trilobed lower lip (Fig. 1). Correspondingly, their styles are generally located in the center of the flower. In addition, the longitudinal anthers, and three yellow spots on the upper side of the corolla tube below the filaments are unique to the species of clades D and E, supporting their sister relationship.

### Ancestral area and character state reconstructions

**Fig. 6:**
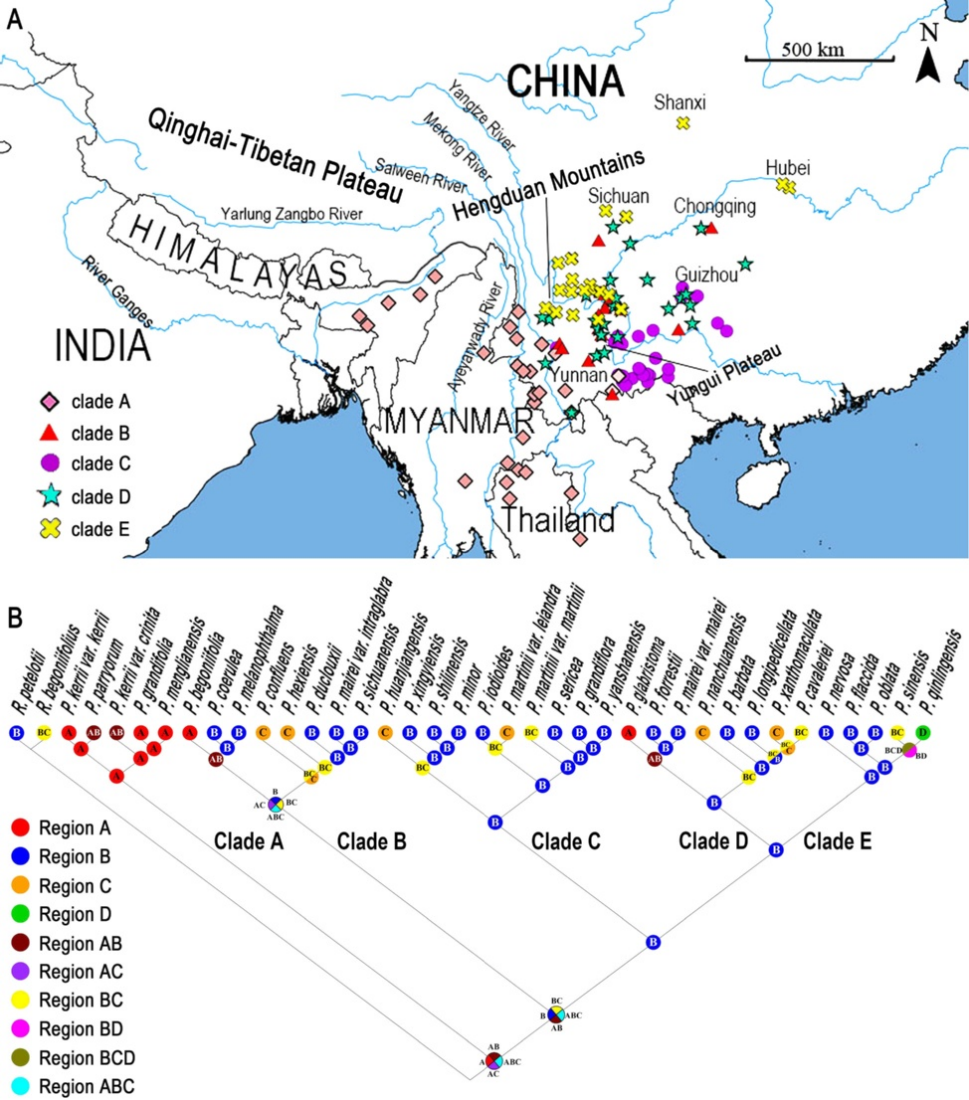
Geographical distribution and ancestral area reconstruction of *Petrocosmea* based on the combined cpDNA and nDNA data. Four areas are defined as follows: Region A, the border region of China, Thailand, India, and Myanmar, lying east and southeast of Himalaya Mountain-Tibetan Plateau; Region B, the Hengduan Mountain-Yunnan Plateau region in southwestern China; Region C, The central China; Region D, the north-central China (only one species, i.e. *Petrocosmea qinlingensis*, belonging to clade E, is distributed in Qinling Mountains (Shanxi province) in north-central China)

The results of ancestral area reconstruction using S-DIVA in RASP is shown in Fig. 6. The most recent common ancestor of *Petrocosmea* is in the border region of China, Thailand, India, and Myanmar, lying east and southeast of Himalaya-Tibetan Plateau. *Petrocosmea* has greatly diversified in southwestern China, especially in Hengduan Mountain-Yungui Plateau region, and further spread to central China (Fig. 6).

**Fig. 7:**
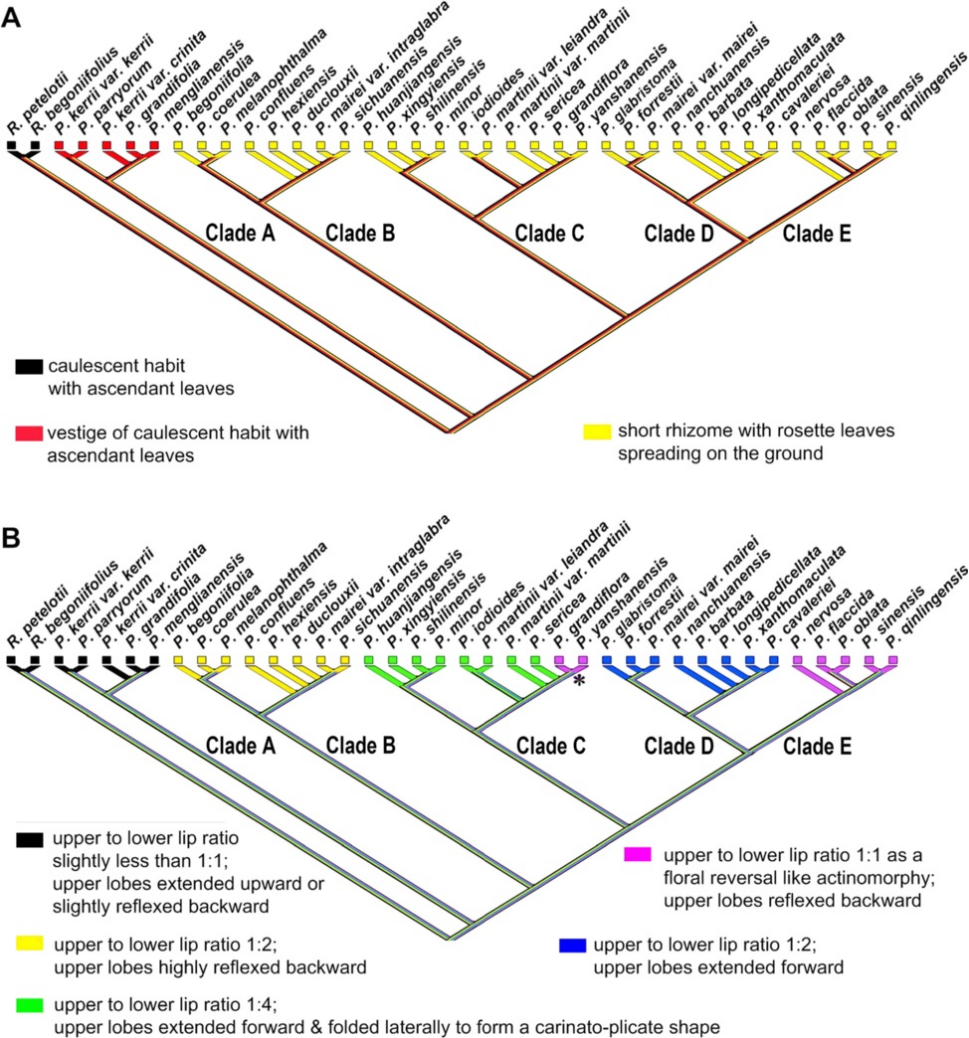
Reconstruction of ancestral states for three morphological characters using Mesquite. Note: An asterisk in the *P. grandiflora*/*P. yanshanensis* branch in clade C indicates a long upper lip but lobed to 1/4 or 1/3 that is distinctive from clade E (B)

**Fig. 8:**
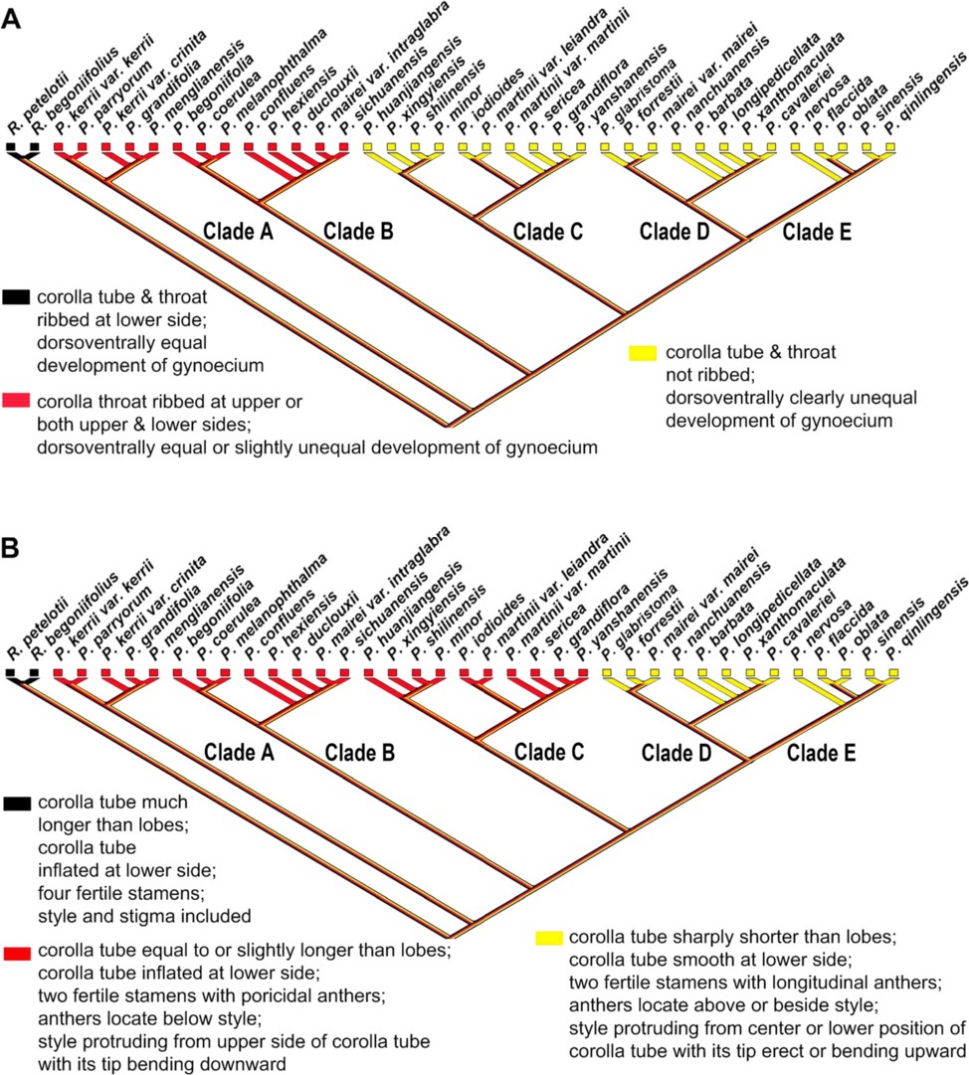
Reconstruction of ancestral states for seven morphological characters using Mesquite

For ancestral character state reconstructions, twelve diagnostic characters were analyzed on the posterior set of trees derived from the combined molecular data analysis (Fig. 2). These were selected among all of the characters that were scored because they may represent important adaptations in the speciation of *Petrocosmea*. They are plant habit, ratio of the upper lip to lower lip, structure of the upper lip; character of corolla throat, dorsoventrally equal/unequal development of the ovary, length ratio of corolla tube to corolla lobes, inflation of the lower part of the corolla tube, position of the anther and filament relative to the ovary and style, type of anther dehiscence, exsertion of the style with curvature type of style tip; constriction at the top of the anther and straight/geniculation of filaments (Figs. 1, 4, 7-8, Additional file 1: Figure S5). We found that the plants of clade A retained a vestigial caulescent habit with ascendant leaves, which transitioned to a habit consisting of a short rhizome with rosette leaves spreading on the ground (Fig. 1). A ratio of upper to lower lip of 1:2 was inferred to have appeared independently two times in clades B and D. The upper lip is reflexed backward in clade B but extended forward in clade D (Figs. 1, 7). The upper to lower lip ratio is 1:4 in the main branch of clade C, but secondarily lengthened to equal length of the lower lip in clade E as well as the *P. grandiflora*/*P. yanshanensis* branch of clade C (Figs. 1, 5, 7). Corolla throat ribbing and whether the gynoecium develops equally or unequally dorsoventrally were correlated in all taxa and character state mapping indicates that a corolla throat that is ribbed on both upper and lower surfaces and a gynoecium that develops only slightly unequally dorsoventrally is the ancestral state for *Petrocosmea* (Fig. 8). Similarly four other characters were correlated; corolla tube length, corolla tube inflation on lower side, number of fertile stamens and type of dehiscence, and exsertion and orientation of the style. The ancestral states for these are a corolla tube that is equal to slightly longer than the lobes, is inflated on the lower surface, two fertile stamens with poricidal dehiscence, and an exserted style that is bent downward (Fig. 8). In clades D and E, the tube is shortened and not inflated and although there are also only two fertile stamens, their dehiscence is longitudinal and the exserted style is bent upward (Fig. 8).

A series of novel morphological traits are correlated with cladogenetic events in *Petrocosmea*. These morphological novelties are mainly reflected in the size and shape of the upper lip. In clade A, the two upper corolla lobes are slightly smaller than the three corolla lobes of the lower lip, generating a moderate floral zygomorphy as in *Raphiocarpus*. In clade B, the two upper corolla lobes are remarkably reduced relative to the three lobes of the lower lip. In clade C, the two much shortened upper corolla lobes are fused and extremely specialized. In clade D, even though the upper lobes are in general similar to those in clade B in size, they are extended forward with a flat face, contrasting with the two upper corolla lobes reflexed backward in clade B. The flowers in clade E are nearly actinomorphic, reflected in the equal length of the upper and lower lips, a deep sinus among the five corolla lobes and a much shortened corolla tube (Fig. 1). These morphological variants in the size and shape of the upper lip are consistent with a series of counterparts in other floral organs, such as character of corolla throat, length ratio of corolla tube to corolla lobes, inflation of the lower part of the corolla tube, position of the anther and filament relative to the ovary and style and type of anther dehiscence, exsertion of the style with curvature type of style tip, and dorsoventrally equal/unequal development of the ovary (Figs. 1, 4, 8).

## Discussion

The monophyly of *Petrocosmea* is well supported by both molecular and morphological data so far as our current sampling is concerned (Figs. 2-3, Additional file 1: Figure S1-S3). The flowers of *Petrocosmea* are characterized by a short corolla tube with a length of only 3-6 mm. This short tube is remarkably different from the flowers of many other Gesneriaceae where corolla tubes often are over 2 cm long [5], but is similar to species of *Saintpaulia* [6]. *Petrocosmea,* with a combination of synapomorphies (perennial stemless herbs, bilateral-diandrous flowers with a short corolla tube and two fertile stamens), is clearly distinguished from its sister group *Raphiocarpus* (subshrub, bilateral-tetrandrous flowers with a long corolla tube over 4 cm long, and four fertile stamens). However, the molecular phylogeny herein does not support the traditional classification of *Petrocosmea* as divided into three sections (*Petrocosmea*, *Anisochilus* and *Deinanthera*). The species of the three sections are scattered across different branches in the phylogenetic trees from all analyses; none of the three sections are recovered as monophyletic regardless of the source of data used for the phylogenetic analyses. In contrast, our molecular data show that *Petrocosmea* consists of five clades corroborated by morphological data as prementioned.

### Origin of *Petrocosmea*

The present molecular phylogeny represents the first major step toward understanding evolution in *Petrocosmea*. Further analyses of morphological characters in light of the molecular phylogeny will enhance our understanding of the morphological origin and diversification in relation to the evolutionary history of this genus. A growing amount of evidence from phylogenetic studies shows that the acaulescent and diandrous-flowered *Petrocosmea* might have proceeded from a caulescent and tetrandrous-flowered *Raphiocarpus*-like ancestor [14, 15]. The tetrandrous flowers with only the mid-upper stamen aborted have been considered an ancestral state in Didymocarpoideae [6, 14, 15]. The morphological evolutionary shift from tetrandrous to diandrous flowers has occurred several times in the Old World Gesneriaceae, such as the shift from tetrandrous *Oreocharis* to *Opithandra* with only two lateral fertile stamens and from tetrandrous *Anna* to diandrous *Lysionotus* with both the mid-upper and lateral stamens aborted [14, 15, 18]. This morphological shift in stamen number usually involves only the increase of sterile stamens from the mid-upper to both the mid-upper and lateral/ventral stamens with the length of corolla tube unchanged in most groups of Gesneriaceae. Therefore, most genera are characterized by a long corolla tube both in tetrandrous and diandrous flowers in Gesneriaceae [5, 6]. However, the flowers of *Petrocosmea* are not only diandrous but also have a short corolla tube of only 3-6 mm as well as acaulescence. The plants of clade A retain a caulescent habit with ascendant leaves. The plants of other clades of *Petrocosmea*, in contrast, are characterized by short rhizomes with leaves spreading on the ground (Fig. 7a). The caulescent habit is correlative with their moderately zygomorphic flowers with a relatively long corolla-tube found in species of clade A that is distinctively different from the strongly zygomorphic flowers and short corolla tube in clades B-D.

Ancestral area reconstruction indicates the origin of *Petrocosmea* in the boundary area of India, Myanmar, Thailand and China, lying east and southeast of Himalaya-Tibetan plateau. This eastern wing region of the Himalaya-Tibetan plateau is one of the most geologically active areas in the world, covered with pure carbonate substrate [19–21]. These limestone areas are characteristic of fluctuating ecological environments with an alternation between severe erosion in the rainy season and extreme drought in the long dry season that are stressful for plant growth [19, 21]. The acaulescent and subsequent rosette habit of *Petrocosmea* might have evolved in response to selective pressures imposed by extreme fluctuation of seasonal climate and ecological conditions. Habitat usually exerts strong influence on vegetative growth adapted for plant survival [22]. However, little is known about the driving force behind the evolution from a long to short corolla tube in the origin of *Petrocosmea.* The shift in corolla tube length is probably related to changes in the insect fauna.

### Functional and evolutionary implications of the lineage-specific morphological differentiation

As outlined above, a series of novel morphological traits are correlated with the cladogenetic events in *Petrocosmea*, all are first documented in present study. They are mainly reflected in the upper lip, i.e. the two upper corolla lobes, in size and shape. Floral zygomorphy (bilateral symmetry) is one key innovation associated with the adaptive radiation of angiosperms because it promotes the coevolution between plants and animals [23–26]. The evolution of floral zygomorphy has been widely considered a major trend in the phylogeny of angiosperms, in which zygomorphy has played a key role in generating the diversity inherent to many large and successful angiosperm clades [23, 26, 27]. Zygomorphy is also found to be one of the three main factors associated with the geographical distribution of diversification hotspots in angiosperms (two other factors are noncontiguous distribution and altitude) [28, 29]. For example, frequent pollinator shifts are correlated with rapid lineage diversification in the flora of southern Africa that has exceptional species richness and endemism [30]. Floral zygomorphy usually promotes reproductive isolation by discrimination in favor of specific pollinators, such as two sympatric species in *Mimulus* marked by different zygomorphic flowers that are specifically pollinated by bees or hummingbirds [31]. In Malpighiaaeae, the floral arrangement rotates 36° between zygomorphic flowers as the pollinator shifts from the oil-bee to xylocopine-bee [32].

In insect-pollinated zygomorphic flowers, the lower lip often functions as a platform for the landing of visiting insects. However, the upper lip plays a key role in attracting or permitting specific pollinators to visit by specialized petal size and shape. Therefore, the lower lip is generally consistent in morphology while the upper lip exhibits variation and specialization in both shape and size [24, 33]. It is especially true for *Petrocosmea*, in which the upper lip tends to be shortened and specialized from clade A through clade B to clade C in size and shape, especially the carinate-plicate shape in clade C, with coordinated variation of correlative characters. Ancestral state and area reconstructions demonstrate that the floral morphological specialization is accompanied by the geographical dispersal from the boundary area of India, Myanmar, Thailand and China, lying east and southeast of Himalaya-Tibetan plateau, to southwestern China, especially the Hengduan Mountain-Yungui plateau areas where *Petrocosmea* is highly diversified. The floral transition from moderate to extremely strong zygomorphy may reflect a pollinator or pollinator-behavior shift likely towards more specialized pollination, which is the major evolutionary trend of the floral zygomorphy in many clades of angiosperms [23, 26, 27]. However, the upper lip demonstrates a reversal from the extremely zygomorphic flowers in clade C through clade D to the almost actinomorphic flowers in clade E, as well as a parallel evolutionary pathway within clade C, correlated with a series of other floral morphological differentiations. This evolutionary reversal also frequently occurs in other groups of Gesneriaceae accompanied by pollinator shifts towards generalized pollination [15]. For example, the tubular zygomorphic flowers specifically pollinated by hummingbirds proceeds to subcampanulate flowers with generalized pollination in Gesnerieae [34], as well as the flat-faced actinomorphic flowers of *Ramonda* evolved from the tubular zygomorphic flowers of *Haberlea* that switched to generalist pollinators [15, 35, 36]. The floral morphological transition with pollinator shift from specialist to generalist is usually related to the evolution of the reproductive assurance mechanism when specialist pollinators are absent or rare [34]. It may apply to the floral evolutionary reversal from clade C to clade E and within clade C in *Petrocosmea*. The lineage-specific differentiation reflected in the upper lip with correlative characters might be related to the evolution of functional morphology to optimize pollination process in the genus *Petrocosmea*. The elaborate morphology of the upper lip characteristic of carinate-plicate shape in clade C may represent a functional innovation for the occurrence of wholly novel or more effective specialist pollinators.

Given that the phenotypic variation in *Petrocosmea* mainly involves floral rather than vegetative traits, the diversification of *Petrocosmea* is likely a product of concerted evolution associated with adaptation to different groups of pollinators rather than a direct response to physical environmental variables. *Petrocosmea* is a mid-sized genus but exhibits extreme diverse floral variants mainly reflected by various forms of the upper lip apparently shaped by different pollinators as shown in other groups of Gesneriaceae. In addition, the floral morphological diversification in *Petrocosmea* involves several evolutionary phenomena, i.e. evolutionary successive specialization from clade A to clade C, reversal from clade C to clade E as well as within clade C, parallel evolution reflected by the parallel branches switching to moderate zygomorphy or almost actinomorphy in clade C, and convergent evolution demonstrated by floral similarity between some branches in clades C and E. These evolutionary phenomena are probably all associated with adaptation to pollination under the background of heterogeneous abiotic and biotic environments in the eastern wing regions of Himalaya-Tibetan plateau. *Petrocosmea* may represent an ideal model for the research of floral evolution related to plant-insect coevolution. Therefore, it merits further study in pollination biology to find whether specific pollinators or pollinator behaviors are responsible for the lineage-specific morphological differentiation, especially for the upper lips, in *Petrocosmea*.

### Utility of morphological characters

According to Craib (1919) [8] and Wang (1985, 1990, 1998) [3, 4, 9], the relative length of the upper and lower lips was the dominant principle to divide sections within *Petrocosmea*. Therefore, all species in clade E and some species in clade C with equal or nearly equal upper and lower lips were grouped in sect. *Petrocosmea*, which are traditionally considered the most primitive in the genus [9]. Most species of clades A, B, C, D with the upper lip shorter than the lower lip were placed in sect. *Anisochilus*, and later, the species of clade A were further identified as the most advanced group due to their unique anther morphology and moved to sect. *Deinanthera* [9].

Some authors hold the opinion that morphological data are problematic in reconstructing phylogenetic trees because morphology is frequently convergent and therefore often misleading [37, 38]. Nevertheless, others argue that morphological data synergistically contribute to phylogenetic trees because of their low intrinsic homoplasy and the problems in resolving homology in morphology can be solved through methodological development and examination using modern tools [39–41]. Abundant morphological characters have been utilized in traditional systematics for more than 200 years, thus the term “morphological feature” is a concept that a lot of people infer as characters that were traditionally used [1]. In this sense it could be difficult to avoid convergence and confusion about different evolutionary status when wholly indiscriminately using them. Relying only on characters that have been used in the traditional classification systems within *Petrocosmea* reveals homoplasy in these traits based on the phylogenetic analyses in this study. However, the same five clades that were recovered based on DNA sequence data were also recovered using a purely morphological data set that utilized numerous character states that had not previously been considered in the classification within *Petrocosmea*, albeit the fewer number of morphological characters did not yield the same level of support for the clades. Our results highlight that morphological features are still important and relevant in resolving phylogenetic relationships, but must be evaluated in concert and not be used in isolation for the "value" of any one character. We here distinguish clade A from other clades based on the retention of a caulescent habit and moderate floral zygomorphy. In contrast to placing them in the same section only based on their similar length of the upper lip in traditional classification, we distinguish the plants of clade B, C (the main branch) and D from each other with character combination of upper to lower lip ration of 1:2 with the upper lip reflexed backward in clade B, upper to lower lip of 1:4 with the upper lip folded laterally to specially form a carinate-plicate shape in the main branch of clade C and upper to lower lip ratio of 1:2 with the upper lip extended forward in clade D. We further interpret the equal length of the upper and lower lip in clade E as well as some branches in clade C as a floral reversal rather than an ancestral feature in the traditional classification according to the molecular phylogeny herein. These recognitions of the major distinctive morphological characters are based on the combination of traditionally used characters and our novel findings in morphology, and reevaluation on the ground of new hypothesis, correlated with a series of other morphological traits. Any one morphological character is not problematic in and of itself but can circumscribe non-monophyletic groups when used in isolation, a priori, as being more useful than other characters.

Traditional investigations of morphology should be renewed or extended in the form of new hypotheses for phylogenetic reconstruction and evolutionary origin. According to Bybee *et al.* (2010) [42], morphological characters were not only infrequently (only 10.5 %) used in recent phylogenetic reconstructions, but the selection of characters were largely unoriginal and untested for their synapomorphies. It is likely that some so-called morphological synapomorphies in traditional systematics are in fact morphological similarities as a result of convergent evolution. Given the large amount of morphological characters utilized in traditional systematics, there is an urgent need to teach old dogs new tricks in present phylogenetic reconstructions.

## Conclusion

In contrast to *Petrocosmea* actually exhibiting extremely diverse floral variation, few morphological characters have been described in the traditional system with poor subgeneric classification. We conduct the first phylogenetic analyses in *Petrocosmea* based on dense taxonomic sampling and multiple loci from two nuclear and six chloroplast DNA regions, which support the monophyly of *Petrocosmea* and recover five major clades within the genus. We further carry out a comprehensive investigation on the flower morphology with living plant material in *Petrocosmea* and find a series of novel morphological traits that are specific to the five respective clades. Reconstructions of ancestral states of twelve morphological characters strongly support five clades revealed by the molecular phylogeny, suggesting these newly observed morphological traits have phylogenetic significance. Phylogenetic analyses and ancestral state reconstructions suggest that the acaulescent *Petrocosmea* with diandrous flowers and short corolla tubes might have proceeded from the caulescent *Raphiocarpus*-like ancestor with tetrandrous flowers and long corolla tubes. Ancestral area reconstruction shows that the geographic origin of *Petrocosmea* lies east and southeast of the Himalaya-Tibetan plateau. Functional and evolutionary analyses of floral morphology indicate that the lineage-specific floral differentiation reflected in the upper lip in *Petrocosmea* are likely adaptive responses to the shift of pollinators or pollinator behaviors, especially the highly specialized structure of the upper lip, a carinate-plicate shape in clade C first recognized herein. We find that the floral morphological diversification in *Petrocosmea* involves several evolutionary phenomena, i.e. evolutionary successive specialization, reversal, parallel evolution, and convergent evolution, which are probably associated with plant-insect coevolution in the heterogeneous abiotic and biotic environments in the eastern wing regions of Himalaya-Tibetan plateau. Further detailed research in pollination biology with ecogeography-associated analyses would shed light on mechanisms underlying the floral evolution and diversity of *Petrocosmea* as an adaptive coevolution responding to local environmental changes. Our results also highlight the importance that morphological features, when evaluated in concert, and through active research to discover new characters, would enhance our understanding of the relationships revealed by molecular phylogeny.

## Methods

### Plant materials

Thirty-five species and three non-nominate varieties including all three sections of *Petrocosmea* sensu Wang (1985) [9] were sampled. Attempts were made to find suitable material of *P. condorensis* Pellegr., *P. kingii* (Clarke) Chatterjee and *P. formosa* B. L. Burtt as well as *P. oblata* var. *latisepala* W. T. Wang, but without success. All materials for DNA extraction came from silica-dried or fresh leaves except for *Petrocosmea confluens* W. T. Wang and *P. grandiflora* Hemsl. for which herbarium specimens at PE and KEW, respectively were used. The voucher information of all sampled taxa and GenBank accession numbers are listed in Additional file 1: Tables S1-S2.

### Outgroup choice for phylogenetic study

To determine the most appropriate outgroup for the phylogenetic study of *Petrocosmea*, a large number of related species from 23 genera of Gesneriaceae were sampled. Twenty genera from other Didymocarpeae, one from Trichosporeae which may be a close relative of Didymocarpeae [12, 13, 43], one from Cyrtandreae, and one from Epithemateae were included. *Antirrhinum majus* (Plantaginaceae) and *Tetranema mexicanum* (Scrophulariaceae) were used as outgroups in preliminary analyses based on *trnL-F* and ITS (Additional file 1: Table S2). The result showed that *Petrocosmea* is well supported as monophyletic and sister to two species of *Raphiocarpus* from Didymocarpeae with strong to moderate support (Additional file 1: Figure S1). Therefore, *Raphiocarpus begoniifolius* and *Raphiocarpus petelotii* were chosen as outgroups with the additional inclusion of one species of *Boea* and two species of *Streptocarpus* for the subsequent phylogenetic analyses in all data sets for this study.

### DNA extraction, PCR amplification, and sequencing for phylogenetic study

Total genomic DNA was extracted from silica-gel-dried, fresh or herbarium specimen leaf materials using the CTAB method of Rogers and Bendich (1988) [44] and used as the templates in the polymerase chain reaction (PCR).

The *atpI-atpH*, *matK*, *trnH-psbA*, *rps16* intron, *trnL-F*, *trnT-L*, and the entire nuclear ribosomal DNA ITS regions were amplified using atpI/atpH [45], matK-AF/trnK-2R [46, 47], trnH/psbA [48, 49], rps16-2 F/rps16-R3 [50], c/f [51], a/b [51], ITS-1/ITS-4 [52], respectively. To amplify the *PeCYC1D* region, a pair of primers were used: forward PD1 (5’–CCC ACA AGA AAT AAT GCT TAG C–3’) and reverse PD2 (5’ – AGC ACA GAT GCC AAA AGA TTC – 3’). The PCR products were purified using Tian quick Midi Purification Kit (Tiangen Biotech, Beijing, China) following the manufacture’s protocol and were directly sequenced. The sequencing primers are the same as amplification primers except *matK* and *PeCYC1D* regions. A reverse primer matK-8R [47] was added in the *matK* region sequencing, and a forward primer F1d (5’–TCA TCC TCC TCA GGT TTC ACA G–3’) and the reverse primer R [18] were used in the *PeCYC1D* sequencing.

### DNA sequence alignment and phylogenetic analyses

Sequences were aligned using Clustal X1.83 [53] and adjusted manually using BioEdit5.0.9 [54]. All combined DNA data were analyzed with maximum parsimony (MP), maximum likelihood (ML) and Bayesian inference (BI) methods, which were implemented in PAUP*^*^4.0b10 [55], RAxML 8.1.11 [56], and MRBAYES version 3.0b4 [57], respectively.

For MP analysis, all characters were given equal weight and character states were unordered. Heuristic searches were performed with 1000 replicates of random addition, one tree held at each step during stepwise addition, tree-bisection-reconnection (TBR) branch swapping, Multrees in effect, and steepest descent off. Bootstrap support [58] for each clade was estimated from 1000 heuristic search replicates as described above.

For ML analysis, the optimal model and parameters were determined under the Akaike information criterion (AIC) in Modeltest 3.06 [59]. A BIONJ tree was employed as a starting point [60]. Statistical support for the node on the ML tree was estimated by 1000 replicates of bootstrap analyses.

In the BI analysis, the model choice of nucleotide substitution was the same as described in ML analysis. Four chains of the Markov Chain Monte Carlo were run each for 10,000,000 generations and were sampled every 10,000 generations. For each run, the first 200 samples were discarded as burn-in to ensure that the chains reached stationary. In the majority rule consensus from Bayesian analysis, posterior probability (PP) was used to estimate robustness.

For combined sequence data, the incongruence length difference (ILD) test [61] as implemented in PAUP* 4.0b10 [55] was performed to assess character congruence between cpDNA data and nDNA data, with 1000 replicates, each with 100 random additions with TBR branch swapping. The p value was used to determine whether the two data sets contained significant incongruence (0.05).

### Constructing a morphological character matrix and reconstructing the ancestral state of some selected morphological characters

The morphological dataset is based on 41 characters, of these, 25 are floral and important traits previously used for subgeneric classification within *Petrocosmea* (see Additional file 1: Appendix S1). The morphological data and the combined matrix of DNA plus morphological data were analyzed with MP and BI methods. The Mk1 model was used for the morphological characters in BI. Characters are equally weighted and the states were unordered.

The evolution of twelve diagnostic characters (for detail see Results) was analyzed on the posterior set of trees from the combined molecular MP analysis. The analysis was performed using unordered maximum parsimony as implemented in Mesquite ver. 3.02 (available from http://mesquiteproject.org). The results are summarized on the majority rule consensus tree of the posterior set of trees.

### Biogeographical analyses

To reconstruct the possible ancestral ranges of *Petrocosmea*, we conducted an S-DIVA analysis [62] using the software package RASP [63]. By utilizing the bootstrap distribution of trees resulting from a MP analysis and generating credibility support values for alternative phylogenetic relationships, the S-DIVA method can minimizes the phylogenetic uncertainties [62, 64, 65].

We used the most parsimonious tree generated from analysis of combined cpDNA and nDNA data as a final representative tree. Four geographic regions were coded: Region A, the border region of China, Thailand, India, and Myanmar, lying east and southeast of Himalaya-Tibetan Plateau; Region B, the Hengduan Mountain-Yunnan Plateau region in southwestern China; Region C, The central China; Region D, the north-central China. By loading the representative tree file and the distribution file based on the geographic region codes as mentioned above, the statistical Dispersal-Vicariance Analysis (S-DIVA) was executed in the software package RASP. Ancestral areas were reconstructed with the “max areas” constrained to three because most species occur in fewer than three areas.

The geographical distribution was generated by ARCGIS 10.2(ESRI,US). Locations of *Petrocosmea* distribution were obtained from collection records and herbarium. The transition from location to longitude and latitude was carried out online (www.gpsspg.com).

### Deposition of phylogenetic data in Treebase

All the phylogenetic data used in this study have been deposited to the Treebase (http://purl.org/phylo/treebase/phylows/study/TB2:S17643).

## Availability of supporting data

The data sets supporting the results of the article are available in GenBank under accession numbers KR006351-KR006603. All of the phylogenetic sequence data in this study are deposited in GenBank (National Center for Biotechnology Information) with the link http://www.ncbi.nlm.nih.gov/nuccore/.

All additional materials supporting the results of the article are included as additional files.

## Additional file

Additional file 1: Figure S1. The strict consensus tree of 1035 MP trees generated from analysis of combined ITS and *trnL-F* DNA sequence data. **Figure S2.** The majority rule consensus Bayesian tree generated from analysis of combined chloroplast DNA regions. **Figure S3.** The majority rule consensus Bayesian tree generated from analysis of combined nuclear DNA regions of ITS and PeCYC1D. **Figure S4.** The strict consensus tree of 15 most parsimonious trees generated from analysis of morphological data. **Figure S5.** Reconstruction of ancestral states for two morphological characters by Mesquite. **Table S1.** Species, voucher with collection locality and GenBank accession number for taxa included for phylogenetic reconstruction in this study. **Table S2.** Species with citation and GenBank accession number for taxa included for the outgroup choice in this study. **Appendix S1.** Morphological characters scored for the phylogenetic analysis.
