# Supplementary material for: Origin and evolution of Petrocosmea (Gesneriaceae) inferred from both DNA sequence and novel findings in morphology with a test of morphology-based hypotheses
Source: BMC Plant Biol. 2015 Jul 3;15:167. doi: 10.1186/s12870-015-0540-3 (PMC4489212; doi:10.1186/s12870-015-0540-3)
Supplement: Additional file 1: Figure S1. — The strict consensus tree of 1035 MP trees generated from analysis of combined ITS and trnL-F DNA sequence data. Figure S2. The majority rule consensus Bayesian tree generated from analysis of combined chloroplast DNA regions. Figure S3. The majority rule consensus Bayesian tree generated from analysis of combined nuclear DNA regions of ITS and PeCYC1D. Figure S4. The strict consensus tree of 15 most parsimonious trees generated from analysis of morphological data. Figure S5. Reconstruction of ancestral states for two morphological characters by Mesquite. Table S1. Species, voucher with collection locality and GenBank accession number for taxa included for phylogenetic reconstruction in this study. Table S2. Species with citation and GenBank accession number for taxa included for the outgroup choice in this study. Appendix S1. Morphological characters scored for the phylogenetic analysis. [file 12870_2015_540_MOESM1_ESM.docx]

**Additional files**

**Figure S1.** The strict consensus of 1035 MP trees (L=3325, CI=0.4, RI=0.693) generated from analysis of combined ITS and *trnL-F* DNA sequence data. MP bootstrap (MP-BS) values ( ≥ 50%) are shown above branches.


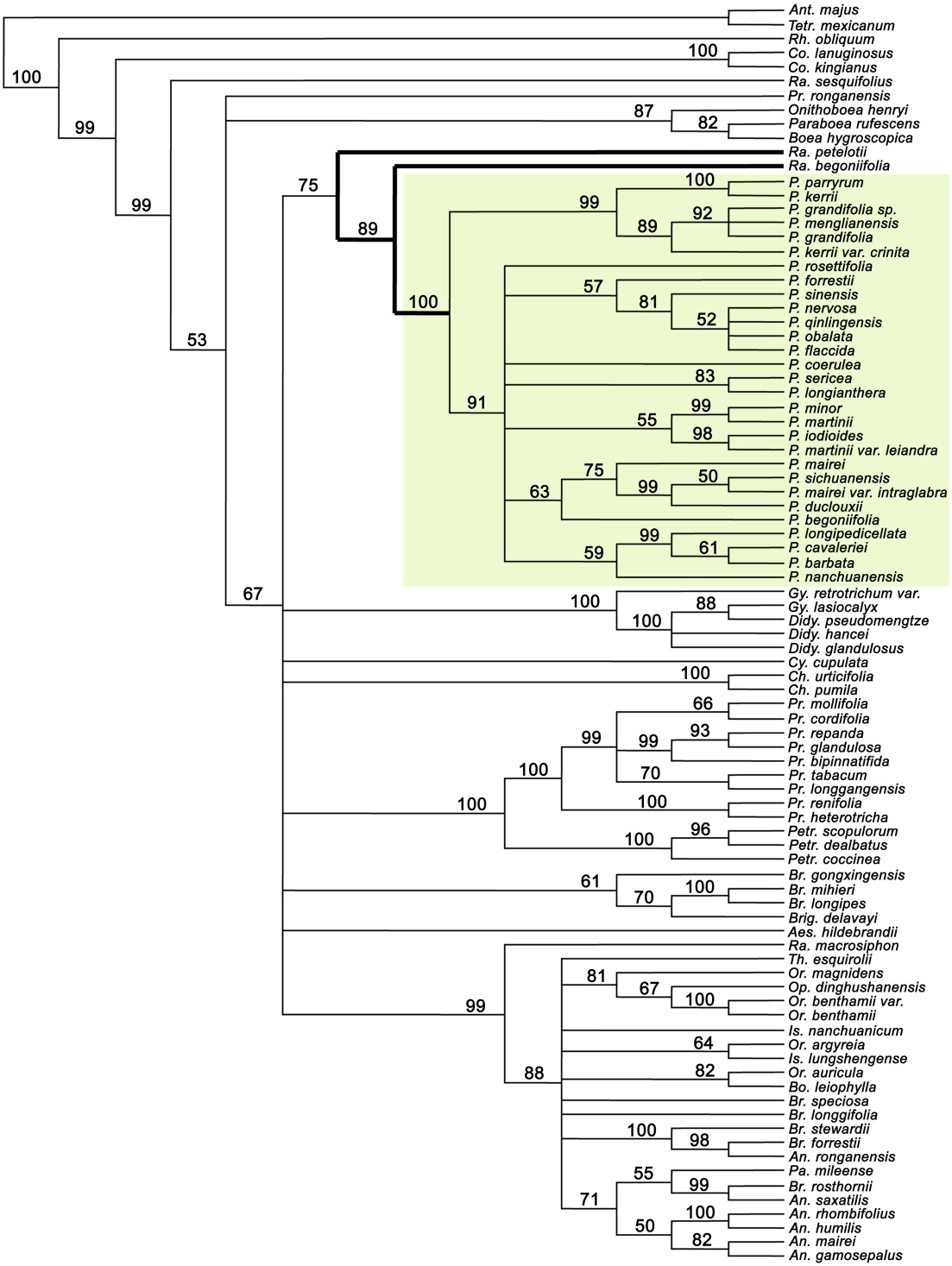


Aes. *Aeschynanthus*; An. *Ancylostemon*; Ant. *Antirrhinum*; Bo. *Bournea*; Br. *Briggsia*; Brig. *Briggsiopsis*; Ch. *Chirita*; Co. *Corallosdiscus*; Cy. *Cyrtandra*; Didy. *Didymocarpus*; Gy. *Gyrocheilos*; Is. *Isometrum*; Op. *Opithandra*; Or. *Oreocharis*; P. *Petrocosmea*; Pa. *Paraisometrum*; Petr. *Petrocodon*; Pr. *Primulina*; Ra. *Raphiocarpus*; Rh. *Rhynchoglossum*; Tetr. *Tetranema*; Th. *Thamnocharis*. Note: the shaded part on the tree indicates all species of the genus *Petrocosmea* grouped together in a single clade.

**Figure S2.** The majority rule consensus Bayesian tree generated from analysis of combined chloroplast DNA regions. Bootstrap (BS) values from MP/ML are shown above branches and posterior probabilities (PP) from BI are shown below branches. P. *Petrocosmea*, R. *Raphiocarpus*, Str. *Streptocarpus*.


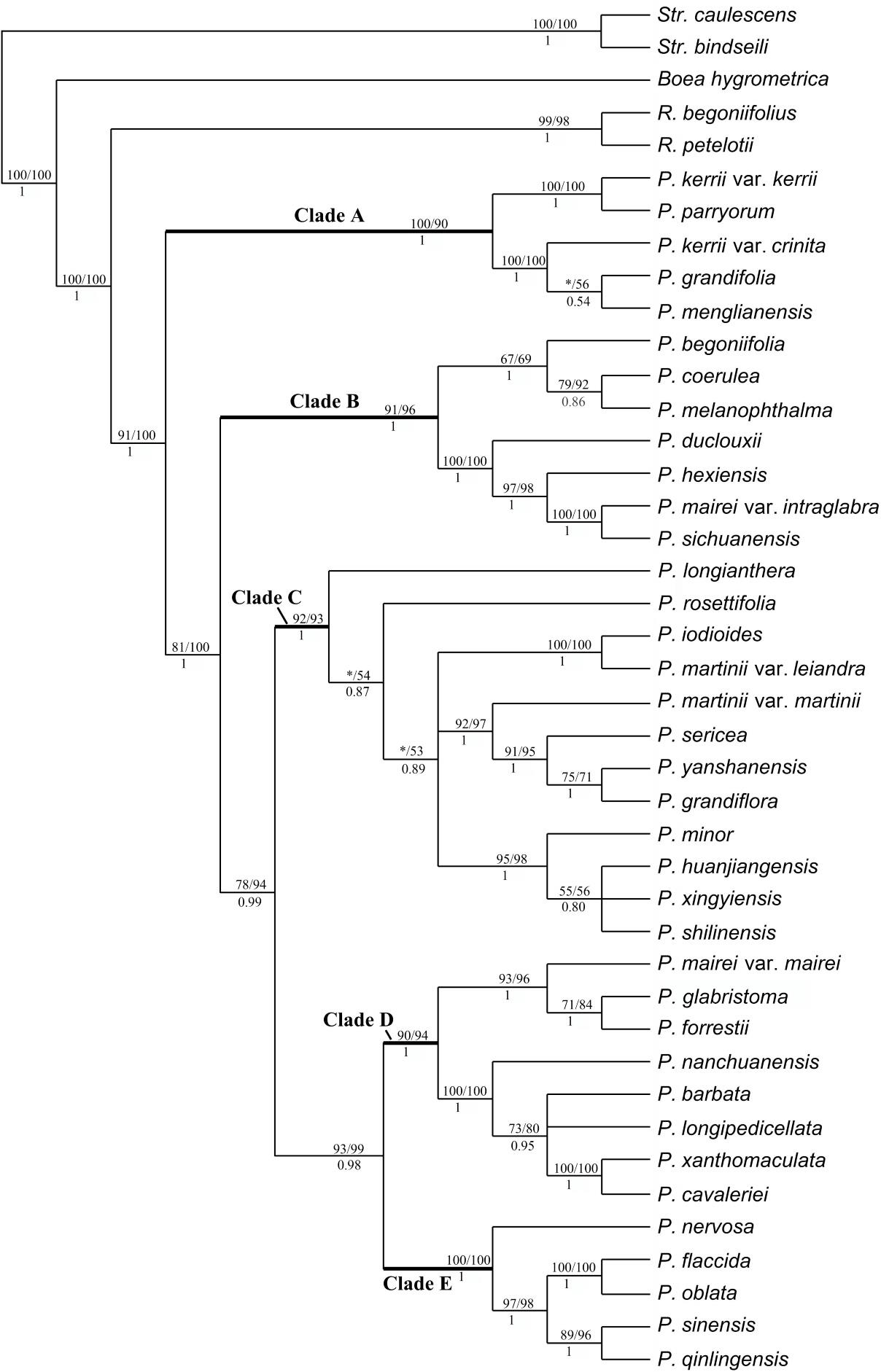


**Figure S3.** The majority rule consensus Bayesian tree generated from analysis of combined nuclear DNA regions of ITS and PeCYC1D. Bootstrap (BS) values from MP/ML are shown above branches and posterior probabilities (PP) from BI are shown below branches. P. *Petrocosmea*, R. *Raphiocarpus*, Str. *Streptocarpus*.


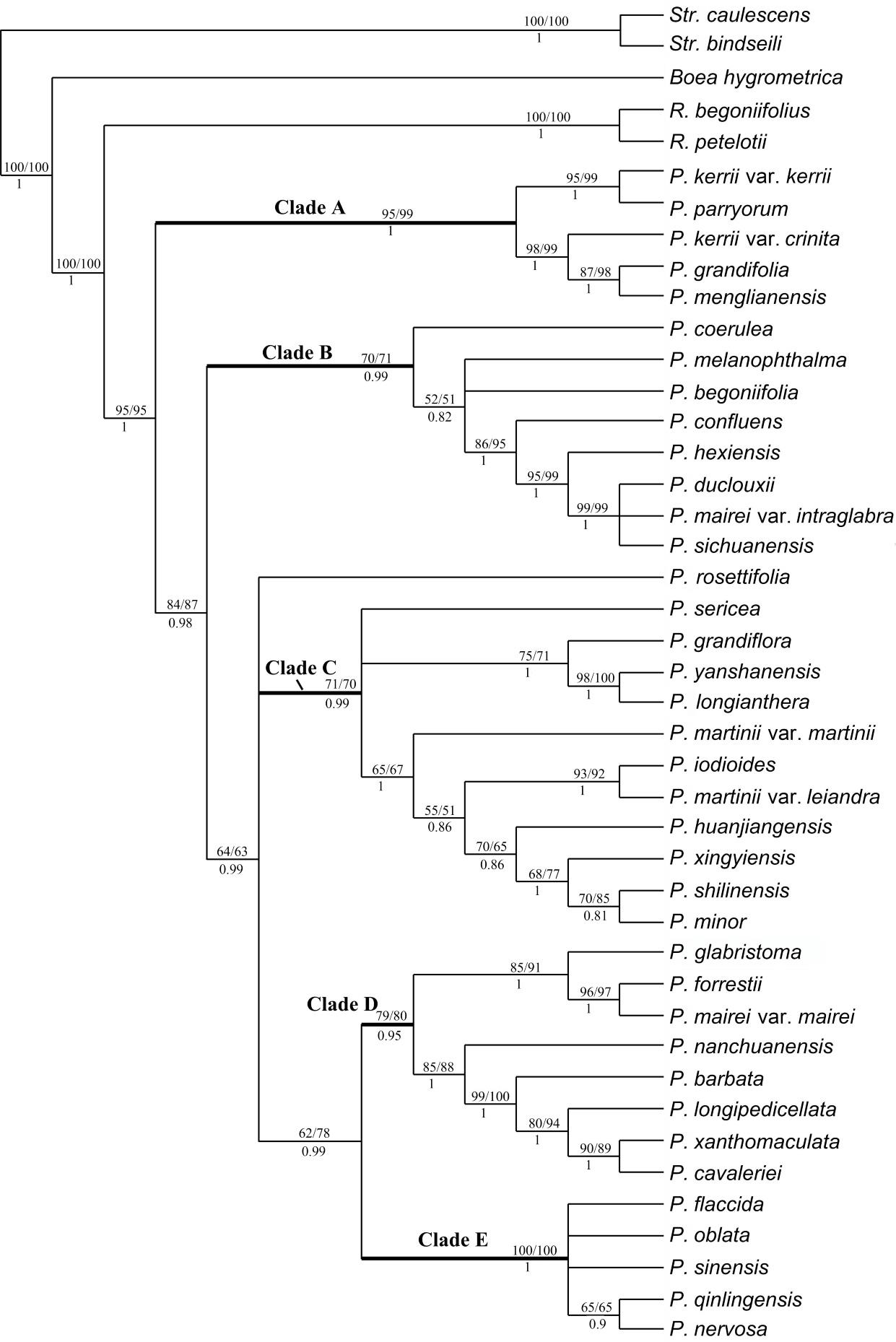


**Figure S4.** The strict consensus tree of 125 most parsimonious trees generated from analysis of morphological data. Bootstrap (BS) values from MP are shown above branches and posterior probabilities (PP) from BI are shown below branches. P. *Petrocosmea*, R. *Raphiocarpus*.

**
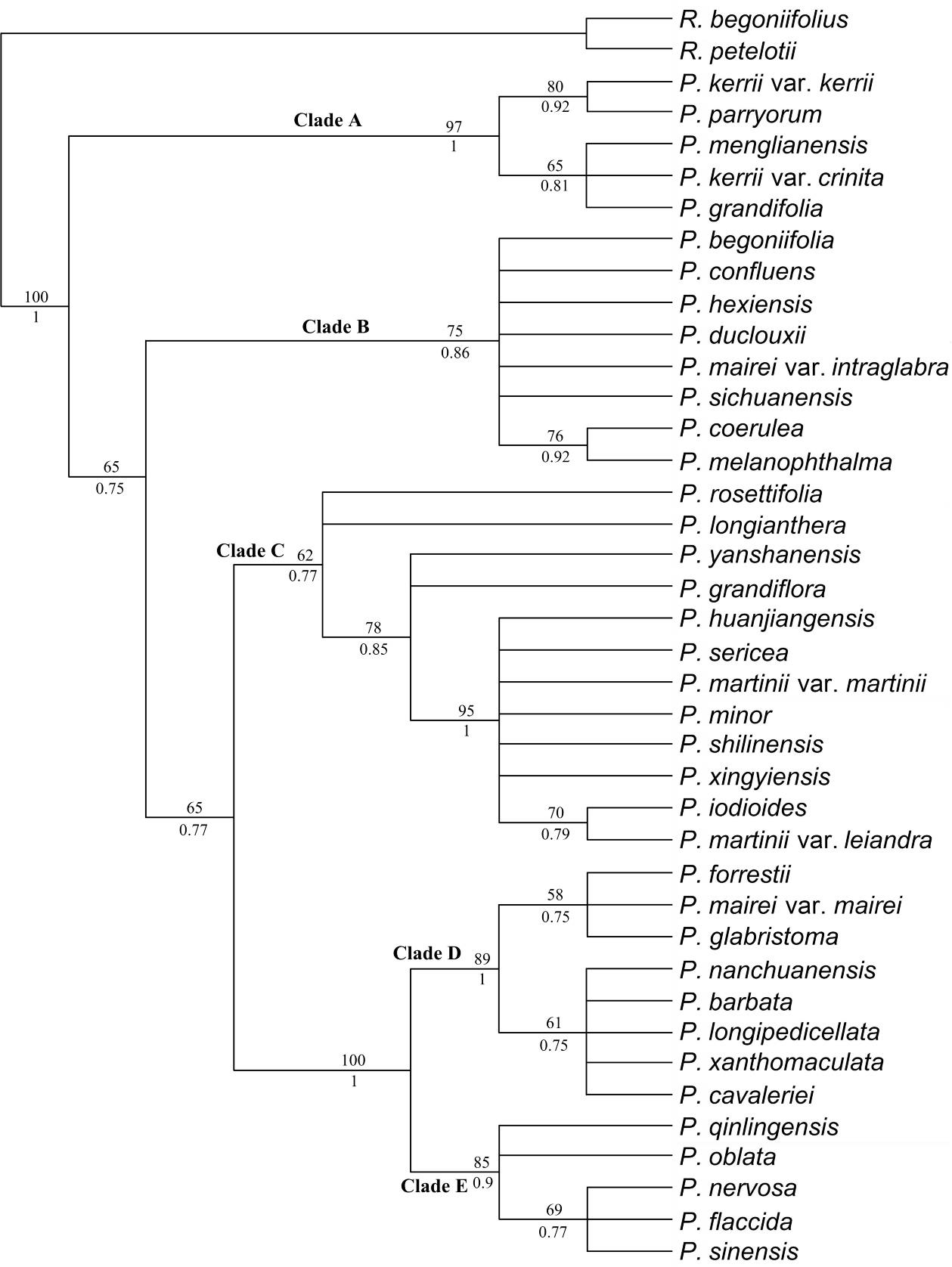
**

**Figure S5.** Reconstruction of ancestral states for two morphological characters by Mesquite.


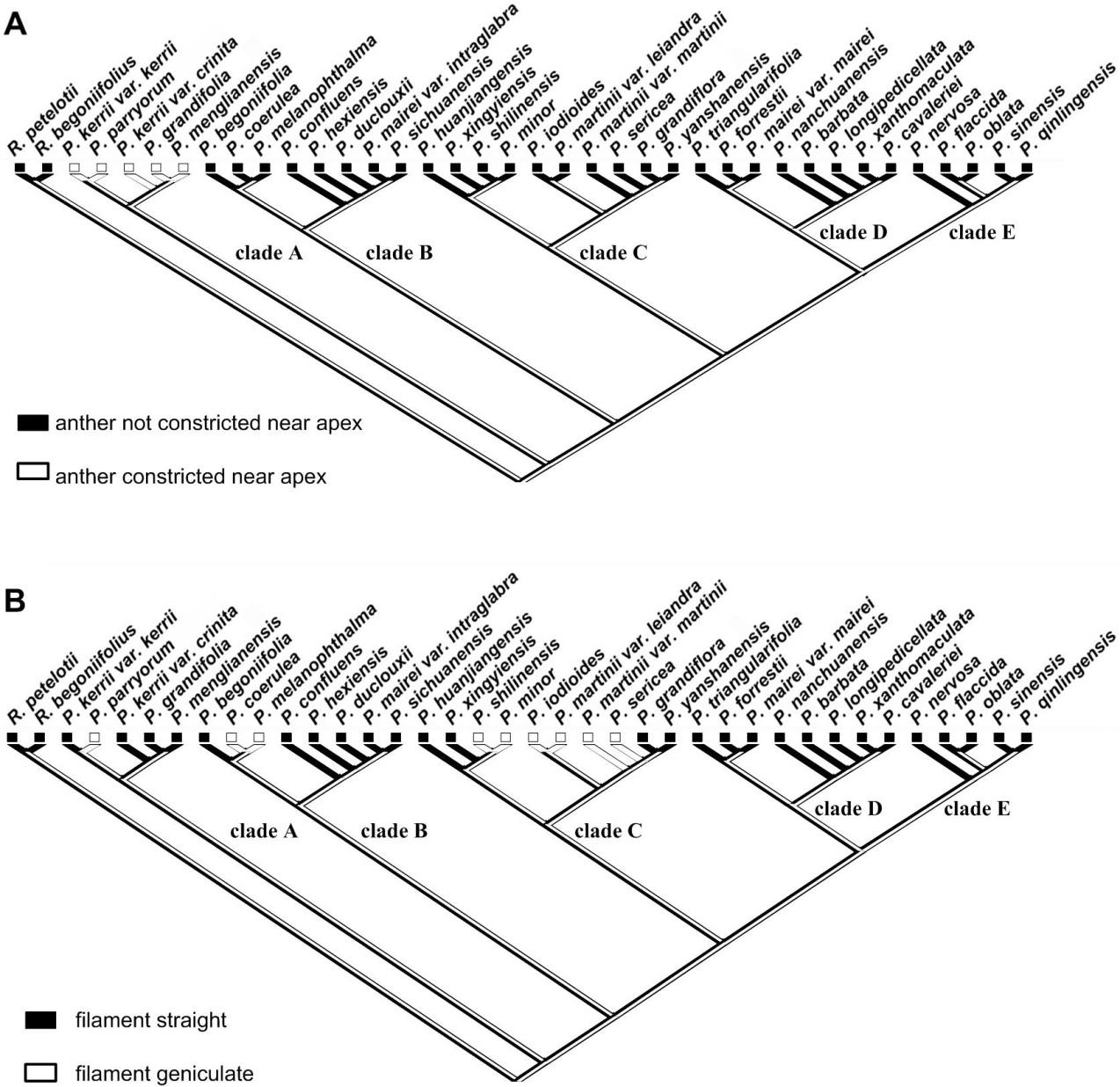


**Table S1.** Species, voucher with collection locality and GenBank accession number for taxa included for phylogenetic reconstruction in this study.

| *Taxon* | *Voucher, collection locality and citation* | **GenBank Accession No.** | | | | | | | |
| --- | --- | --- | --- | --- | --- | --- | --- | --- | --- |
|  |  | *trnL-F* | *matK* | *rps16* | *psbA-trnH* | *atpI-H* | *trnT-L* | ITS | *PCyc1D* |
| **Ingroups** |  |  |  |  |  |  |  |  |  |
| **Gesneriaceae** |  |  |  |  |  |  |  |  |  |
| *Petrocosmea barbata* Craib | QZJ-2007-009, Yunnan, China (PE^*^) | KR006351 | KR006438 | KR006491 | KR006402 | KR006555 | KR006424 | KR006475 | KR006583 |
| *Petrocosmea begoniifolia* C. Y. Wu ex H. W. Li | QZJ-2007-051, Yunnan, China (PE) | KR006361 | KR006456 | KR006494 | KR006397 | KR006546 | KR006431 | KR006482 | KR006564 |
| *Petrocosmea cavaleriei* H. Lévl. | QZJ-2007-082, Guizhou, China (PE) | KR006372 | KR006440 | KR006487 | KR006403 | KR006553 | KR006420 | KR006476 | KR006581 |
| *Petrocosmea coerulea* C. Y. Wu ex W. T. Wang | 991000, Yunnan, China (KUN^**^) | KR006355 | KR006457 | KR006492 | KR006406 | KR006552 | KR006427 | KR006483 | KR006571 |
| *Petrocosmea confluens* W. T. Wang | 1385, Guizhou, China (PE) | ― | ― | ― | ― | ― | ― | KR006466 | KR006569 |
| *Petrocosmea duclouxii* Craib | Q06100101, Yunnan, China (PE) | KR006360 | KR006458 | KR006498 | KR006388 | KR006543 | KR006433 | KR006478 | KR006568 |
| *Petrocosmea flaccida* Craib | Q060921-1, Sichuan, China (PE) | KR006363 | KR006443 | KR006517 | KR006379 | KR006533 | KR006414 | KR006471 | KR006592 |
| *Petrocosmea forrestii* Craib | QZJ-2008-58, Yunnan, China (PE) | KR006365 | KR006445 | KR006520 | KR006380 | KR006532 | KR006416 | KR006464 | KR006595 |
| *Petrocosmea glabristoma* Z. J. Qiu & Y. Z. Wang*.* | QZJ-2007-061, Yunnan, China (PE) | KR006362 | KR006444 | KR006516 | KR006377 | KR006531 | KR006417 | KR006468 | KR006591 |
| *Petrocosmea grandiflora* Hemsl. | Hancock 115, Yunnan, China (K^***^) | KR006373 | KR006454 | KR006504 | KR006409 | ― | KR006437 | KR006467 | KR006574 |
| *Petrocosmea grandifolia* W. T. Wang | QZJ-2007-037, Yunnan, China (PE) | JN092472 | JN092541 | KR006507 | KR006393 | KR006548 | JN092505 | JN092439 | KR006588 |
| *Petrocosmea hexiensis* S. Z. Zhang & Z. Y. Liu | Z.Y. Liu 110128, Chongqing, China (SZG^****^) | KR006359 | KR006461 | KR006497 | KR006386 | KR006559 | KR006415 | KR006469 | KR006567 |
| *Petrocosmea huangjiangensis* Yan Liu & W. B. Xu | QZJ-1344, Guangxi, China (SZG) | KR006367 | KR006448 | KR006503 | KR006398 | KR006561 | KR006435 | KR006484 | KR006576 |
| *Petrocosmea iodioides* Hemsl. | QZJ-2007-074, Yunnan, China (PE) | JN092473 | JN092542 | KR006513 | KR006390 | KR006539 | JN092506 | JN092440 | KR006601 |
| *Petrocosmea kerrii* Craib | 04603, Yunnan, China (KUN) | JN092474 | JN092543 | KR006509 | KR006395 | KR006551 | JN092507 | JN092441 | KR006584 |
| *Petrocosmea kerrii* Craib var. *crinita* W. T. Wang | QZJ-2007-084, Yunnan, China (PE) | KR006353 | KR006452 | KR006505 | KR006391 | KR006549 | KR006425 | KR006462 | KR006587 |
| *Petrocosmea longianthera* Z. J. Qiu & Y. Z. Wang | QZJ-2007-079, Yunnan, China (PE) | JN092448 | JN092517 | KR006514 | KR006407 | KR006541 | JN092481 | JN092398 | KR006572 |
| *Petrocosmea longipedicellata* W.T.Wang | QZJ-2007-083, Yunnan, China (PE) | KR006352 | KR006439 | KR006490 | KR006404 | KR006556 | KR006422 | KR006474 | KR006579 |
| *Petrocosmea mairei* H. Lévl. | 019140, Yunnan, China (KUN) | KR006366 | KR006447 | KR006519 | KR006381 | KR006537 | KR006418 | KR006465 | KR006598 |
| *Petrocosmea mairei* H.Lévl. var. *intraglabra* W. T. Wang | QZJ-2007-068, Yunnan, China (PE) | KR006357 | KR006460 | KR006495 | KR006385 | KR006545 | KR006430 | KR006479 | KR006570 |
| *Petrocosmea martinii* H. Lévl. | QZJ-2007-078, Yunnan, China (PE) | JN092475 | JN092544 | KR006501 | KR006408 | KR006538 | JN092508 | JN092442 | KR006577 |
| *Petrocosmea martinii* var. *leiandra* W. T. Wang | QZJ-2008-33, Guizhou, China (PE) | JN092476 | JN092545 | KR006512 | KR006389 | KR006540 | JN092509 | JN092443 | KR006599 |
| *Petrocosmea melanophthalma* Huan C. Wang, Z. R. He & Li Bing Zhang | QZJ-1409, Yunnan, China (SZG) | KR006356 | KR006449 | KR006493 | KR006399 | KR006558 | KR006436 | KR006481 | KR006582 |
| *Petrocosmea menglianensis* H. W. Li | QZJ-2007-026, Yunnan, China (PE) | JN092477 | JN092546 | KR006506 | KR006394 | KR006547 | JN092510 | JN092444 | KR006586 |
| *Petrocosmea minor* Hemsl. Hook. | QZJ-2008-54, Yunnan, China (PE) | JN092478 | JN092547 | KR006515 | KR006384 | KR006526 | JN092511 | JN092445 | KR006600 |
| *Petrocosmea nanchuanensis sp. nov.* | 2002016, Chongqing, China (PE) | KR006371 | KR006442 | KR006489 | KR006405 | KR006557 | KR006423 | KR006472 | KR006597 |
| *Petrocosmea nervosa* Craib | QZJ-2008-45, Sichuan, China (PE) | JN092479 | JN092548 | KR006523 | KR006382 | KR006536 | JN092512 | JN092446 | KR006590 |
| *Petrocosmea oblata* Craib | Q060923-1, Sichuan, China (PE) | GU350692 | JN092549 | KR006518 | KR006378 | KR006530 | JN092513 | GU350661 | KR006596 |
| *Petrocosmea parryorum* C. E. C. Fisch. | 050801, Yunnan, China (KUN) | KR006354 | KR006453 | KR006508 | KR006392 | KR006550 | KR006426 | KR006463 | KR006585 |
| *Petrocosmea qinlingensis* W. T. Wang | QZJ-2008-38, Shanxi, China (PE) | KR006364 | KR006446 | KR006521 | KR006375 | KR006534 | KR006419 | KR006470 | KR006594 |
| *Petrocosmea rosettifolia* C. Y. Wu ex H. W. Li | QZJ-2007-049, Yunnan, China (PE) | KR006369 | KR006455 | KR006510 | KR006396 | KR006542 | KR006428 | KR006473 | KR006589 |
| *Petrocosmea sericea* C. Y. Wu ex H. W. Li | 991104, Yunnan, China (KUN) | JN092467 | JN092536 | KR006500 | KR006411 | KR006528 | JN092500 | JN092434 | KR006565 |
| *Petrocosmea shilinensis* Y. M. Shui & H. T. Zhao | QZJ-1333, Yunnan, China (SZG) | KR006370 | KR006450 | KR006511 | KR006401 | KR006560 | KR006434 | KR006486 | KR006578 |
| *Petrocosmea sichuanensis* Chun ex W. T. Wang | Q060912-1, Sichuan, China (PE) | KR006358 | KR006459 | KR006496 | KR006387 | KR006544 | KR006429 | KR006480 | KR006566 |
| *Petrocosmea sinensis* Oliver | QZJ-2008-41, Sichuan, China (PE) | GU350691 | JN092550 | KR006522 | KR006376 | KR006535 | JN092514 | GU350660 | KR006593 |
| *Petrocosmea xanthomaculata* G. Q. Gou et X. Y. Wang | QZJ-1077, Guizhou, China (SZG) | KR006374 | KR006441 | KR006488 | KR006400 | KR006554 | KR006421 | KR006477 | KR006580 |
| *Petrocosmea xingyiensis Y. G. Wei & F. Wen* | QZJ-1337, Guizhou, China (SZG) | KR006368 | KR006451 | KR006502 | KR006383 | KR006527 | KR006432 | KR006485 | KR006575 |
| *Petrocosmea yanshanensis* Z. J. Qiu & Y. Z. Wang | QZJ-2007-077, Yunnan, China (PE) | JN092462 | JN092531 | KR006499 | KR006410 | KR006529 | JN092495 | JN092429 | KR006573 |
| **Outgroups** |  |  |  |  |  |  |  |  |  |
| *Raphiocarpus begoniifolius* (Lévl) Burtt | QZJ-2008-026, Guizhou, China (PE) | GU350680 | JN092551 | KR006524 | KR006412 | KR006562 | JN092515 | GU350648 | KR006602 |
| *Raphiocarpus petelotii* (Pellegr) Burtt | GX_NP_1, Guangxi, China (PE) | JN092480 | JN092552 | KR006525 | KR006413 | KR006563 | JN092516 | JN092447 | KR006603 |
| *Boea hygrometrica* (Bunge.) R. Br. | [1, 2~~]~~ | FJ501476 | NC_016468 | NC_016468 | NC_016468 | NC_016468 | NC_016468 | FJ501319 | ― |
| *Streptocarpus bindseili* Eb. Fisch. | [3, 4, 5] | FN794074 | AF531810 | FN794111 | ― | ― | ― | AF316960 | ― |
| *Streptocarpus caulescens* Vatke | [4, 6] | AJ430918 | AJ429331 | AJ431043 | ― | ― | ― | AF316920 | ― |

^*^PE: Herbarium, Institute of Botany, Chinese Academy of Sciences; ^**^KUN: Herbarium, Kunming Institute of Botany, Chinese Academy of Sciences; ^***^K: Herbarium, Kew Royal Botanic Gardens; ^****^SZG: Herbarium, Fairylake Botanical Garden, Shenzhen & Chinese Academy of Sciences where the voucher specimens were deposited.

**References**

1. Möller M, Pfosser M, Jang CG, Mayer V, Clark A, Hollingsworth ML, Barfuss MHJ, Wang YZ, Kiehn M, Weber A: **A preliminary phylogeny of the ‘didymocarpoid Gesneriaceae’ based on three molecular data sets Incongruence with available tribal classifications.** *Amer. J. Bot.* 2009, **96**: 989-1010.

2. Zhang T, Zhang X, Hu S, Yu J: **An efficient procedure for plant organellar genome assembly, based on whole genome data from the 454 GS FLX sequencing platform.** *Plant Methods* 2011, **7** (1): 38.

3. Schaeferhoff K, Michalakis S, Tanimoto N, Fischer MD, Becirovic E, Beck SC, Huber G, Rieger N, Riess O, Wissinger B, Biel M, Seeliger MW, Bonin M: **Induction of STAT3-related genes in fast degenerating cone photoreceptors of cpfl1 mice.** *Cell Mol. Life Sci.* 2010, **67**(18):3173-3186.

4. Möller M, Cronk QCB: Evolution of morphological novelty: **A phylogenetic analysis of growth patterns in Streptocarpus (Gesneriaceae).** *Evolution* 2001, **55**: 918-929.

5. Muller K, Borsch T, Legendre L, Porembski S, Theisen I, Barthlott W: **Evolution of carnivory in Lentibulariaceae and the Lamiales.** *Plant Biol.* 2004, **6** (4): 477-490.

6. Bremer B, Bremer K, Heidari N, Erixon P, Olmstead RG, Anderberg AA, Källersjö M, Barkhordarian E: **Phylogenies of asterids based on 3 coding and 3 non-coding chloroplast DNA markers and the utility of non-coding DNA at higher taxonomic levels.** *Mol. Phylogenet. Evol.* 2002, **24**: 273-301.

**Table S2.** Species with citation and GenBank accession number for taxa included in the outgroup choice in this study.

| *Taxon* | *citation* | **GenBank Accession No.** | | |
| --- | --- | --- | --- | --- |
|  |  | *trnL-F* | ITS |  |
| **Species for outgroup choice** |  |  |  |  |
| **Ingroups** |  |  |  |  |
| **Gesneriaceae** |  |  |  |  |
| *Aeschynanthus hildebrandii* Hemsl. ex Hook.f. | [1] | AY047099 | AY047040 |  |
| *Ancylostemon aureus* (Franch.) B.L.Burtt | [2] | GU350688 | GU350657 |  |
| *Ancylostemon humilis* W. T. Wang | [2] | GU350665 | GU350633 |  |
| *Ancylostemon mairei* (Levl.) Craib | [2] | GU350689 | GU350658 |  |
| *Ancylostemon rhombifolius* K. Y. Pan | [2] | GU350664 | GU350632 |  |
| *Ancylostemon ronganensis* K. Y. Pan | [3] | HQ632927 | HQ633023 |  |
| *Ancylostemon saxatilis* (Hemsl.) Craib | [4] | HQ327451 | HQ327466 |  |
| *Boea hygroscopica* F. Muell | [5] | FJ501477 | FJ501320 |  |
| *Bournea leiophylla* W. T. Wang et K. Y. Pan ex W. T. wang | [2] | GU350676 | GU350644 |  |
| *Briggsia dongxingensis* Chun ex K. Y. Pan | [2] | GU350686 | GU350655 |  |
| *Briggsia kurzii* (C.B.Clarke) W.E.Evans | [3] | HQ632874 | HQ632970 |  |
| *Briggsia longifolia* Craib | [3] | HQ632934 | HQ633030 |  |
| *Briggsia longipes* (Hemsl. ex Oliv.) Craib | [2] | GU350684 | GU350653 |  |
| *Briggsia mihieri* (Franch.) Craib | [2] | GU350678 | GU350646 |  |
| *Briggsia rosthornii* (Diels) B. L. Burtt | [5] | FJ501547 | FJ501365 |  |
| *Briggsia muscicola* (Diels) Craib | [5]; | FJ501548 | FJ501366 |  |
| *Briggsia stewardii* Chun | [3] | HQ632926 | HQ633022 |  |
| *Briggsiopsis delavayi* (Franch.) K. Y. Pan | [2] | GU350679 | GU350647 |  |
| *Calcareoboea coccinea* C. Y. Wu ex H. W. Li | [5] | FJ501516 | FJ501341 |  |
| *Primulina heterotricha* (Merr.) Y.Dong & Yin Z.Wang | [6] | DQ872816 | DQ872826 |  |
| *Primulina longgangensis* (W.T.Wang) Y.Z.Wang | [6] | DQ872809 | DQ872833 |  |
| *Henckelia pumila* (D. Don) A.Dietr. | [6] | DQ872819 | DQ872836 |  |
| *Primulina ronganensis* (D. Fang & Y. G. Wei) Mich.Moeller & A.Weber | [7] | JX506833 | JX506942 |  |
| *Henckelia urticifolia* (D.Don.) A.Dietr. | [6] | DQ872821 | DQ872835 |  |
| *Primulina bipinnatifida* (W.T.Wang) Y. Z.Wang & J. M. Li | [6] | DQ872806 | DQ872842 |  |
| *Primulina cordifolia* (D.Fang & W.T.Wang) Yin Z.Wang | [6] | DQ872803 | DQ872845 |  |
| *Primulina glandulosa* (D.Fang, L.Zeng & D.H.Qin) Yin Z.Wang | [6] | DQ872804 | DQ872841 |  |
| *Primulina mollifolia* (D.Fang & W.T.Wang) J.M.Li & Y. Z.Wang | [6] | DQ872802 | DQ872847 |  |
| *Primulina repanda* (W.T.Wang) Yin Z.Wang | [7, 8] | AJ492292 | JX506941 |  |
| *Corallodiscus kingianus* (Craib) B.L.Burtt | [2] | GU350663 | GU350630 |  |
| *Corallodiscus lanuginosus* (Wall. ex DC.) B.L.Burtt | [2] | GU350662 | GU350631 |  |
| *Cyrtandra cupulata* Ridl. | [8] | AJ492273 | AY818826 |  |
| *Didymocarpus stenanthos* Clarke | [5, 9] | FJ501512 | DQ912687 |  |
| *Didymocarpus hancei* Hemsl. | [2] | GU350667 | GU350667 |  |
| *Didymocarpus pseudomengtze* W.T.Wang | [2] | GU444002 | GU444003 |  |
| *Gyrocheilos lasiocalyx* W.T.Wang | [3] | HQ632901 | HQ632998 |  |
| *Gyrocheilos retrotrichum* W.T.Wang | [3] | HQ632903 | HQ632999 |  |
| *Isometrum lungshengense* (W.T.Wang) W.T.Wang & K.Y.Pan | [2] | GU350690 | GU350659 |  |
| *Isometrum primuliflorum* (Batalin) B.L.Burtt | [3] | HQ632923 | HQ633019 |  |
| *Opithandra dinghushanensis* W.T.Wang | [2] | GU350675 | GU350643 |  |
| *Oreocharis argyreia* Chun ex K.Y.Pan | [2] | GU350671 | GU350639 |  |
| *Oreocharis auricula* (S.Moore) C.B.Clarke | [2] | GU350670 | GU350638 |  |
| *Oreocharis benthamii* C.B.Clarke | [2] | GU350674 | GU350642 |  |
| *Oreocharis argyreia* Chun ex K.Y. Pan var. *angustifolia* K.Y. Pan | [2] | GU350671 | GU350639 |  |
| *Oreocharis magnidens* Chun ex K.Y.Pan | [2] | GU350673 | GU350641 |  |
| *Ornithoboea arachnoidea* (Diels) Craib | [10] | JN934709 | JN934751 |  |
| *Paraboea rufescens* (Franch.) B.L.Burtt | [2] | DQ872825 | DQ865196 |  |
| *Paraisometrum mileense* W.T.Wang | [3] | JF697587 | JF697575 |  |
| *Petrocodon dealbatus* Hance | [2] | GU350668 | GU350636 |  |
| *Primulina tabacum* Hance | [5, 8] | AJ492300 | FJ501352 |  |
| *Raphiocarpus macrosiphon* (Hance) B.L.Burtt | [2] | GU350685 | GU350654 |  |
| *Raphiocarpus sesquifolius* (C.B.Clarke) B.L.Burtt | [3] | HQ632911 | HQ633007 |  |
| *Rhynchoglossum obliquum* Blume | [2] | GU350683 | GU350652 |  |
| *Petrocodon scopulorus* (Chun) Yin Z.Wang | [2] | GU350669 | GU350637 |  |
| *Thamnocharis esquirolii* (H.Lev.) W.T.Wang | [2] | GU350677 | GU350645 |  |
| *Primulina renifolia* (D.Fang & D.H.Qin) J.M.Li & Yin Z.Wang | [2] | GU350669 | GU350637 |  |
| **Outgroup** |  |  |  |  |
| **Scrophulariaceae** |  |  |  |  |
| *Antirrhinum majus* L. | [8, 11] | AJ492270 | AF513888 |  |
| *Tetranema mexicanum* Benth | [8, 12] | AJ492272 | AF375151 |  |

**References**

1. Zimmer EA, Roalson EH, Skog LE, Boggan JK, Idnurm A: **Phylogenetic relationships in the Gesnerioideae (Gesneriaceae) based on nrDNA ITS and cpDNA trnL-F and trnE-T spacer region sequences.** *Am. J. Bot.* 2002, **89**(2): 296–311.

2. Wang YZ, Liang RH, Wang BH, Li JM, Qiu ZJ, Li ZY, Weber A: **Origin and phylogenetic relationships of the Old World Gesneriaceae with actinomorphic flowers inferred from ITS and *trnL-trnF* sequences.** *Taxon* 2010, **59** (4): 1044-1052.

3. Möller M, Forrest A, Wei YG, Weber A: **A molecular phylogenetic assessment of the advanced Asiatic and Malesian didymocarpoid Gesneriaceae with focus on non-monophyletic and monotypic genera.** *Plant Syst. Evol.* 2011, **292**: 223–248

4. Tan Y, Wang Z, Sui X, Hu G: **The systematic placement of the monotypic genus *Paraisometrum* (Gesneriaceae) based on molecular and cytological data.** *Plant Diversity and Resources* 2011, **33**(5): 465-476.

5. Möller M, Pfosser M, Jang CG, Mayer V, Clark A, Hollingsworth ML, Barfuss MHJ, Wang YZ, Kiehn M, Weber A: **A preliminary phylogeny of the ‘didymocarpoid Gesneriaceae’ based on three molecular data sets Incongruence with available tribal classifications.** *Amer. J. Bot.* 2009, **96**: 989-1010.

6. Li JM, Wang YZ: **Phylogenetic reconstr uction among species of *Chiritopsis* and *Chirita* sect. *Gibbosaccus* (Gesneriaceae) based on nrDNA I TS and cpDNA trnL-F sequences.** *Systematic Botany* 2007, **32**: 888–898.

7. Kang M, Tao J, Wang J, Ren C, Qi Q, Xiang QY, Huang H: **Adaptive and nonadaptive genome size evolution in Karst endemic flora of China.** *New Phytol.* 2014, **202** (4): 1371-1381.

8. Mayer V, Möller M, Perret M, Weber A: **Phylogenetic position and generic differentiation of Epithemateae (Gesneriaceae) inferred from plastid DNA sequence data.** *Amer. J. Bot.* 2003, **90**: 321-329.

9. Palee P, Denduangboripant J, Anusarnsunthorn V, Möller M: **Molecular phylogeny and character evolution of *Didymocarpus* (Gesneriaceae) in Thailand.** *Edinburgh Journal of Botany.* 2006, **63**: 231–251.

10. Puglisi C, Middleton DJ, Triboun P, Möller M: **New insights into the relationships between *Paraboea*, *Trisepalum*, and *Phylloboea* (Gesneriaceae) and their taxo-nomic consequences.** *Taxon* 2011, **60**: 1693-1702.

11. Oyama RK, Baum DA: **Phylogenetic relationships of North American Antirrhinum (Veronicaceae).** *American J. Botany* 2004, **91**(6): 918-925.

12. Wolfe AD, Datwyler SL, Randle CP: **A phylogenetic and biogeographic analysis of the *Cheloneae* (Scrophulariaceae) based on ITS and matK sequence data.** *Syst. Bot.* 2002, **27** (1): 138-148.

**Appendix S1.** Morphological characters scored for the phylogenetic analysis

**1.** Leaves : ascendant (0), rosette spreading on the ground (1);

**2.** Are there aerial stems: yes (0), no (1);

**3.** Is there glandular hairs on the leaves: no (0), yes (1);

**4.** Is there disc in a flower: yes (0), no (1);

**5.** The symmetry of calyx: actinomorphy (0), zygomorphy (1);

**6.** The type of calyx: campanulate (0), upper three sepals slightly connate (1), holoblastic (2);

**7.** The situation of small protrusions inside the dorsal corolla tube: no protrusion (0), longitudinal protrusions at the opening (1);

**8.** The situation of small protrusions inside the ventral corolla tube: no protrusion (0), widely longitudinal protrusions at the opening (1), longitudinal protrusions at the opening (2);

**9.** The length ratio of corolla tube to corolla lips: 4-5 (0), ≈1 (1), >1 (2), <1 (3);

**10.** Spots inside the throat of corolla tube: purple and white stripes or two purple spots (0), no spot (1), two yellow spots (2), two slight white (3);

**11.** Spots under the filament in the corolla tube: purple and white stripes (0), two dark red brown spots (1), two purple spots (2), two brown spots (3), no spot (4);

**12.** Spots or color inside the lower side of corolla tube: no stripes (0), white or with white stripes (1);

**13.** Spots inside the upper side of corolla tube: no spots (0), three yellow spots (1);

**14.** The split degree of upper lip: bipartite (0), near to the middle (1), indistinctly two-lobed (2), more than middle (3), near to the base (4);

**15.** Shape of the corolla upper lip: flat (0), reflexed backward extremely (1), laterally folded (2), lean forward (3), reflexed backward slightly (4);

**16.** The ratio of width/length of dorsal corolla lobes: <1 (0), ≈1 (1), >1 (2);

**17.** The ratio of width/length of lateral corolla lobes: ≈1 (0), <1 (1), >1 (2);

**18.** The ratio of width/length of ventral corolla lobes: ≈1 (0), <1 (1), >1 (2);

**19.** Inflation of the lower part of the corolla tube: yes (0), no (1);

**20.** Coat of the inner surface of corolla tube: glabrous or sparsely short pubescent (0), densely pubescent (1);

**21.** Coat of the inner surface of upper corolla lip: glabrous (0), sparsely short pubescent (1), densely pubescent (2);

**22.** Ratio of upper to lower lip: slightly less than 1;1 (0), 1:2 (1), 1:4 (2), 1:1 (3);

**23.** The number of stamens: four (0), two (1);

**24**. Whether constricted at the top of the anther: yes (0), no (1);

**25.** Whether convergent of anther chambers: yes (0), no (1);

**26.** The positional relationship of the anther and filament: anther under the filament (0), anther beside or above the filament (1);

**27.** Whether the stamens are coalescent together: yes (0), no (1);

**28.** Dehiscence type of the anther: longitudinally dehiscent (0), poricidal (1);

**29.** Anther attached on the filament: dorsifixed (0), basifixed (1);

**30.** Parallel or constricted of the two anther chambers: parallel or nearly parallel (0), constricted from the base to top (1);

**31.** Inflection of the filament at the middle: no (0), yes (1);

**32.** Filaments are curve or not: not curve (0), geniculation (1), curve (2);

**33.** Ovary hair: glabrous (0), densely short pubescent and glandular haired (1), densely spreading villous and glandular haired (2), expanded densely pubescent and short glandular haired (3);

**34.** Style hair: glabrous or nearly glabrous (0), sparsely glandular haired (1), densely spreading villous and glandular haired at the middle and base (2), pubescent and glandular haired at the base (3);

**35.** The number of stigma: two (0), one (1),;

**36.** Curved downward of the style base: no (0), yes (1);

**37.** The position of style out of the corolla tube: not out (0), above (1), middle or under middle (2);

**38.** Curvature types of the style tip: not curved (0), slightly downward (1), slightly upward (2);

**39.** The way of style out of the ovary: gradually (0), suddenly (1);

**40.** Whether equally developed between dorsal and ventral locules: yes (0), no (1);

**41.** Is style closes to the dorsal edge of corolla tube and enclasped by the upper corolla lip: no (0), yes (1).
